# miR-93/miR-106b/miR-375-CIC-CRABP1: a novel regulatory axis in prostate cancer progression

**DOI:** 10.18632/oncotarget.4372

**Published:** 2015-06-08

**Authors:** Nahyun Choi, Jongmin Park, Jeon-Soo Lee, Jeehyun Yoe, Guk Yeol Park, Eunjeong Kim, Hyeongrin Jeon, Yong Mee Cho, Tae-Young Roh, Yoontae Lee

**Affiliations:** ^1^ Department of Life Sciences, Pohang University of Science and Technology, Pohang, Kyungbuk, Republic of Korea; ^2^ Division of Integrative Bioscience and Biotechnology, Pohang University of Science and Technology, Pohang, Kyungbuk, Republic of Korea; ^3^ Department of Pathology, University of Ulsan College of Medicine, Asan Medical Center, Seoul, Republic of Korea

**Keywords:** prostate cancer, capicua, ETV5, CRABP1, microRNA

## Abstract

Capicua (CIC) has been implicated in pathogenesis of spinocerebellar ataxia type-1 (SCA1) neurodegenerative disease and some types of cancer; however, the role of CIC in prostate cancer remains unknown. Here we show that CIC suppresses prostate cancer progression. CIC expression was markedly decreased in human prostatic carcinoma. CIC overexpression suppressed prostate cancer cell proliferation, invasion, and migration, whereas CIC RNAi exerted opposite effects. We found that knock-down of CIC derepresses expression of *ETV5* and *CRABP1* in LNCaP and PC-3 cells, respectively, thereby promoting cell proliferation and invasion. We also discovered that miR-93, miR-106b, and miR-375, which are known to be frequently overexpressed in prostate cancer patients, cooperatively down-regulate CIC levels to promote cancer progression. Altogether, we suggest miR-93/miR-106b/miR-375-CIC-CRABP1 as a novel key regulatory axis in prostate cancer progression.

## INTRODUCTION

CIC is an HMG box-containing transcriptional repressor evolutionarily conserved from nematodes to humans [1]. CIC preferentially binds to TG/CAATGA/GA sequences within promoters and enhancers of target genes in *Drosophila* and mammals [2], and a bacterial one-hybrid screen for DNA binding motifs of *Drosophila* transcription factors has revealed that the consensus sequence of CIC binding motifs is 5′-YYCATTSA-3′ [3, 4]. At least two CIC isoforms exist in *Drosophila* and mammals, CIC-L and CIC-S, which differ in their amino-terminal regions. The longer isoform CIC-L contains a unique amino-terminal region of approximately 900 amino acids in length in mammals [2]. In mammals, CIC was identified as an interacting protein of ATXN1, the causative protein of SCA1 neurodegenerative disease [5]. Haploinsufficiency of CIC partially rescues ataxia phenotypes in *Atxn1^154Q^* knock-in mice, suggesting that CIC facilitates pathogenesis of SCA1 [6]. It is also known that *Cic* hypomorphic (*Cic-L^−/−^*) mice have defects in lung alveolarization and bile acid homeostasis [7, 8].

Several previous findings have implicated that CIC might be involved in tumorigenesis and/or cancer progression in humans. First, dozens of mutations in *CIC* have been identified in patients with various types of cancers [9-11]. Second, a chromosomal translocation generating a CIC-DUX4 fusion was identified in Ewing-like sarcomas [12]. Third, the best known target genes of CIC include *PEA3* group genes, *ETV1*/*ER81*, *ETV4*/*PEA3*, and *ETV5*/*ERM*, which are frequently overexpressed in several different types of cancers [13, 14]. Despite the findings supporting the potential role of CIC in cancer, it has not been clear whether CIC deficiency or mutations indeed contribute to cancer progression. In this study, we show that CIC functions as a negative regulator of prostate cancer progression.

## RESULTS

### CIC is down-regulated in prostate cancer cells

**Figure 1:**
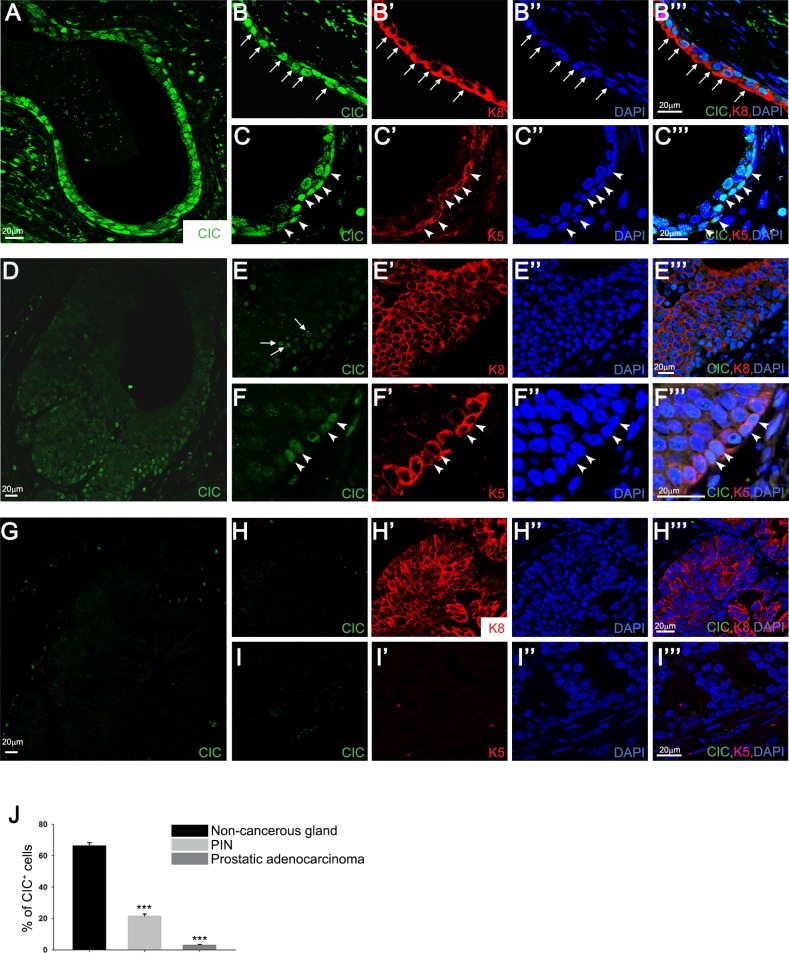
Decreased nuclear expression of CIC in prostate cancer cells **A.-C.**”’ Immunocytochemistry for CIC, keratin 8 (K8, luminal cell marker), and keratin 5 (K5, basal cell marker) in prostate cancer patient specimens. CIC is markedly expressed in the nucleus of non-cancerous prostate glands. Arrows and arrow heads indicate luminal and basal cells, respectively. Images were taken from the specimen of patient #3. **D.-F.**”’ Expression patterns of CIC in prostatic intraepithelial neoplasia (PIN). The number of cells with the nuclear expression of CIC was apparently reduced in PIN compared with the non-cancerous prostate glands. Arrows and arrow heads indicate luminal and basal cells, respectively. Images were taken from the specimen of patient #3. **G.-I.**”’ Absence of nuclear expression of CIC in prostatic adenocarcinoma. Images were taken from the specimen of patient #2. **J.** Quantitative analysis for the proportion of cells with nuclear expression of CIC in non-cancerous prostate glands, PIN, and prostatic adenocarcinoma. Four to seven regions of non-cancerous prostate glands, PIN, and prostatic adenocarcinoma per each specimen were randomly selected from all the tested patient samples, and the proportion of nuclear CIC signal-positive cells was examined. ***P < 0.001. All error bars show s.e.m.

Given that several ETS transcription factor genes (*ERG* and *PEA3* group genes) are frequently overexpressed due to chromosomal translocations in prostate cancer cells, thereby contributing to prostate cancer pathogenesis [15], we hypothesized that CIC might suppress prostate cancer progression through repressing expression of *PEA3* group genes. To test this hypothesis, we first examined expression of CIC in mouse prostate cells by immunocytochemistry. We found that CIC is expressed in the nucleus of both basal and luminal cells of mouse prostate glands (Supplementary Figure 1). As a control, a marked decrease in fluorescence signal in thymus sections from *Cic-L*^−/−^ mice [6, 7] compared with wild-type (WT) littermates verified the suitability of our antibody [8] in evaluating CIC expression (Supplementary Figure 2). We next determined patterns of CIC expression in prostate cancer patient specimens. Consistent with the results from mouse (Supplementary Figure 1), CIC is apparently expressed in the nuclei of both basal and luminal cells of non-cancerous human prostate glands (Figures 1A∼1C”’). Intriguingly, however, the proportion of cells with nuclear CIC expression was markedly decreased in prostatic intraepithelial neoplasia (PIN) (Figures 1D∼1F”’). Moreover, the nuclear expression of CIC disappeared in advanced prostatic adenocarcinoma with complete loss of basal cells (Figures 1G∼1I”’). These results were consistently observed in prostate tissue specimens from 13 different prostate cancer patients (Supplementary Table 1) and the average values for the proportion of cells with nuclear CIC expression in non-cancerous prostate glands, PIN, and prostatic adenocarcinoma are presented in Figure 1J. Consistent with these findings, CIC protein levels were lower in prostate cancer cell lines than in PNT2 normal prostate epithelial cell (Supplementary Figure 3). Notably, CIC levels were the lowest in PC-3, the most aggressive type of prostate cancer cell [16], among the tested prostate cell lines (Supplementary Figure 3). Taken together, these data demonstrate that prostate cancer progression is accompanied with down-regulation of CIC expression.

### CIC suppresses cell proliferation, invasion and migration in prostate cancer cells

**Figure 2:**
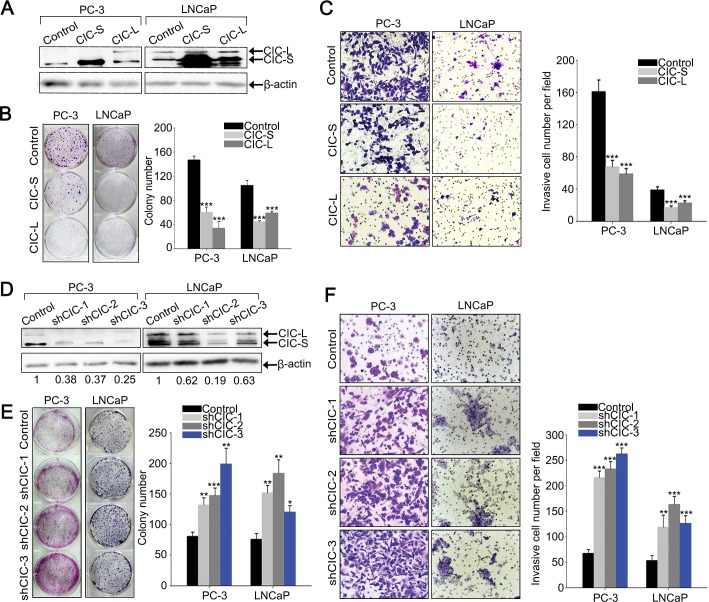
CIC suppresses cell proliferation and invasion in PC-3 cells **A.** Western blot analysis for ectopic expression of CIC-S and CIC-L in PC-3 and LNCaP cells. **B.** Clonogenic assay showing inhibition of cell growth by overexpression of CIC in PC-3 and LNCaP cells. The right panel is a bar graph for quantitative analysis on colony numbers. Three independent experiments were performed. ***P < 0.001. All error bars show s.e.m. **C.** Matrigel invasion assay showing inhibition of cell invasion by overexpression of CIC in PC-3 and LNCaP cells. The right panel is a bar graph for quantitative analysis on invasive cell numbers. Three independent experiments were performed. ***P < 0.001. All error bars show s.e.m. **D.** Western blot analysis to examine CIC knock-down efficiency of three different shRNAs against *CIC* (shCIC-1∼3) in PC-3 and LNCaP cells. Relative CIC levels were calculated based on band intensities of CIC and β-actin, and indicated below the images. Two and three independently obtained western blot images for samples of PC-3 and LNCaP cells, respectively, were subjected to the quantitative analysis for the relative CIC levels. **E.** Clonogenic assay showing promotion of cell growth by knock-down of CIC in PC-3 and LNCaP cells and its quantification. Three independent experiments were performed. *P < 0.05, **P < 0.01 and ***P < 0.001. All error bars show s.e.m. **F.** Matrigel invasion assay showing promotion of cell invasion by knock-down of CIC in PC-3 and LNCaP cells and its quantification. Three independent experiments were performed. **P < 0.01 and ***P < 0.001. All error bars show s.e.m.

We then examined whether the decrease in CIC levels is necessary for promotion of prostate cancer progression. We overexpressed CIC in PC-3 and LNCaP cells by infection with lentivirus expressing either mouse CIC-S or CIC-L (Figure 2A), and checked cell proliferation, invasion, and migration. Clonogenic and BrdU labeling assays demonstrated that overexpression of CIC suppresses prostate cancer cell proliferation (Figures 2B and Supplementary Figure 4). Moreover, cell invasion and migration were markedly inhibited in PC-3 and LNCaP cells overexpressing CIC (Figure 2C and Supplementary Figure 5A). We also tested whether deficiency of CIC could promote prostate cancer progression. To this end, we generated prostate cancer cell lines that stably express three different shRNAs targeting *CIC* (shCIC-1∼3). These CIC shRNAs showed different knock-down efficiency in each cell line: shCIC-3 most dramatically decreased CIC levels in PC-3, while such was the case for shCIC-2 in LNCaP (Figure 2D). Both clonogenic and BrdU labeling assays demonstrated that reduction in CIC levels significantly increases cell proliferation in PC-3 and LNCaP cells (Figure 2E and Supplementary Figure 6). We also found that invasive property of cells was markedly enhanced by knock-down of CIC in both LNCaP and PC-3 cells (Figure 2F) and that cell migration was significantly increased in the CIC knock-down PC-3 cells (Supplementary Figure 5B). The increases in cell proliferation, invasion, and migration were apparently correlated with CIC knock-down efficiency, suggesting that these results were certainly due to a decrease in CIC levels and not owing to the off-target effect of CIC shRNAs. Taken together, these data demonstrate that CIC could function as a negative regulator in prostate cancer progression.

### Derepression of *ETV5* contributes to the increases in cell proliferation and invasion in the CIC knock-down LNCaP cells

**Figure 3:**
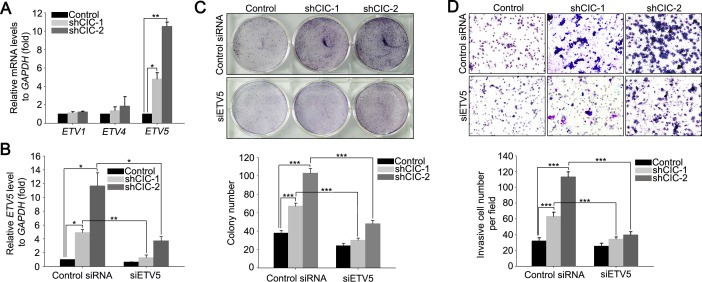
Derepression of *ETV5* contributes to the increased cell proliferation and invasion in CIC knock-down LNCaP cells **A.** qRT-PCR analysis for *ETV1*, *ETV4* and *ETV5* levels in the CIC knock-down LNCaP cells. Three independent experiments were performed. *P < 0.05 and **P < 0.01. All error bars show s.e.m. **B.** qRT-PCR analysis for *ETV5* levels in the CIC knock-down LNCaP cells transfected with either control or *ETV5* siRNA. Three independent experiments were performed. *P < 0.05 and **P < 0.01. All error bars show s.e.m. **C.** Clonogenic assay using control, shCIC-1 and shCIC-2 LNCaP cells treated with either control or *ETV5* siRNA. Suppression of *ETV5* expression reduced colony formation in the shCIC-1 and shCIC-2 LNCaP cells. The lower panel is a bar graph for quantitative analysis on colony numbers. Three independent experiments were performed. ***P < 0.001. All error bars show s.e.m. **D.** Matrigel invasion assay using control, shCIC-1 and shCIC-2 LNCaP cell lines treated with either control or *ETV5* siRNA. Suppression of *ETV5* expression reduced the number of invasive cells. The lower panel is a bar graph for quantitative analysis on invasive cell numbers. Three independent experiments were performed. ***P < 0.001. All error bars show s.e.m.

Given that *PEA3* group genes are regulated by CIC and that altered expression of these genes is associated with the pathogenesis of various types of cancers including prostate cancer [17], we assessed levels of *PEA3* group genes in the CIC knock-down cell lines by qRT-PCR. Significant up-regulation of *ETV5* levels was found in CIC knock-down LNCaP cells (Figure 3A), while unexpectedly, levels of *ETV1*, *ETV4*, and *ETV5* were comparable among control and CIC knock-down PC-3 cell lines (Supplementary Figure 7), suggesting a cell-type specific regulation of *PEA3* group genes by CIC. We then examined whether the derepression of *ETV5* contributed to promotion of cell proliferation and invasion in the CIC knock-down LNCaP cells. We treated the shCIC-1 and shCIC-2 LNCaP cells with siRNA against *ETV5* (siETV5) to make the level of *ETV5* similar to that in control cells (Figure 3B), and assessed cell proliferation and invasion. The RNAi against *ETV5* significantly suppressed cell proliferation and invasion in the CIC knock-down LNCaP cells (Figures 3C and 3D and Supplementary Figure 8), indicating that *ETV5* is a critical target of CIC in LNCaP cells in terms of regulation of cancer progression.

### Overexpression of *CRABP1* contributes to the increases in cell proliferation and invasion in the CIC knock-down PC-3 cells

**Figure 4:**
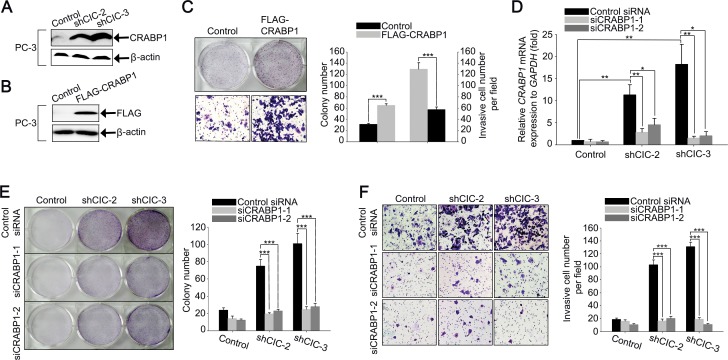
Overexpression of CRABP1 promotes cancer progression in CIC knock-down PC-3 cells **A.** Western blot analysis for endogenous CRABP1 expression in control and CIC knock-down PC-3 cell lines. **B.** Western blot analysis for ectopic expression of FLAG-CRABP1 in PC-3 cells. **C.** Clonogenic and matrigel invasion assays showing promotion of cell growth and invasion, respectively, by overexpression of CRABP1 in PC-3 cells, and its quantification. Three clonogenic assays and five matrigel invasion assays were performed independently. ***P < 0.001. All error bars show s.e.m. **D.** qRT-PCR analysis for *CRABP1* levels to check suppression of *CRABP1* expression by siRNAs against *CRABP1* (siCRABP1-1 and siCRABP1-2) in control and CIC knock-down PC-3 cell lines. Seven independent experiments were performed. *P < 0.05 and **P < 0.01. All error bars show s.e.m. **E.** Clonogenic assay using control and CIC knock-down PC-3 cell lines treated with two different *CRABP1* siRNAs. Suppression of *CRABP1* expression reduced colony formation in the CIC knock-down PC-3 cell lines. The right panel is a bar graph for quantitative analysis on colony numbers. Four independent experiments were performed. ***P < 0.001. All error bars show s.e.m. **F.** Matrigel invasion assay using control and CIC knock-down PC-3 cell lines treated with two different *CRABP1* siRNAs. Suppression of *CRABP1* expression reduces the number of invasive cells. The right panel is a bar graph for quantitative analysis on invasive cell numbers. Three independent experiments were performed. ***P < 0.001. All error bars show s.e.m.

Given the comparable expression of *PEA3* group genes in control and CIC knock-down PC-3 cells (Supplementary Figure 7), we investigated which gene expression changes upon knock-down of CIC promoted cell proliferation and invasion in PC-3 cells. To this end, we analyzed gene expression profiles in control and CIC knock-down (shCIC-3) PC-3 cells. The high throughput mRNA sequencing analysis revealed that a total of 262 genes (159 up-regulated, and 103 down-regulated) was differentially expressed (Fold change (log2) > 2 and P-value < 0.05) in the shCIC-3 PC-3 cells compared with control cells (Supplementary Table 2). Among the differentially expressed genes, cellular retinoic acid binding protein 1 (*CRABP1*) primarily caught our attention, because of the highest fold increase upon knock-down of CIC in PC-3 cells (Supplementary Table 2) and its previously known pro-tumorigenic and pro-metastatic activity in mesenchymal tumors [18]. Moreover, it is known that *CRABP1* is overexpressed in castration-resistant prostate cancer cells [19]. Consistent with this, we observed that *CRABP1* levels are much higher in PC-3 cells, which are independent of androgen for their growth [16], than in LNCaP and PNT2 cells (Supplementary Figure 9). We confirmed the overexpression of CRABP1 at protein level in the CIC knock-down PC-3 cells by western blot analysis (Figure 4A). To verify that the overexpression of *CRABP1* was certainly due to deficiency of CIC, we carried out RNAi against *CIC* in PC-3 cells using siRNA duplexes (siCIC) and examined the expression of *CRABP1* by qRT-PCR. The treatment with siCIC also up-regulated *CRABP1* levels in PC-3 cells (Supplementary Figure 10), suggesting that CIC indeed negatively regulates *CRABP1* expression. On the other hand, we found that *CRABP1* levels were not significantly altered in CIC knock-down LNCaP cells (Supplementary Figure 11), indicating that CIC regulates *CRABP1* expression in a cell-type dependent manner.

We then explored whether CRABP1 could regulate cell proliferation and invasion in prostate cancer cells. Lentivirus-mediated overexpression of FLAG-CRABP1 increased cell proliferation as well as invasion in PC-3 cells (Figures 4B and 4C and Supplementary Figure 12), suggesting that CRABP1 can function as a positive regulator of prostate cancer progression. Finally, we examined whether the up-regulation of *CRABP1* levels contributed to the increased cell proliferation and invasion in the CIC knock-down PC-3 cells. We carried out RNAi against *CRABP1* using two different *CRABP1* siRNAs (siCRABP1-1 and siCRABP1-2) in the CIC knock-down PC-3 cells to make the level of *CRABP1* comparable to that in control cells (Figure 4D). Under this condition, we performed clonogenic, cell growth, and invasion assays, and found that transfection with *CRABP1* siRNAs reduced cell proliferation and invasion in the CIC knock-down PC-3 cells (Figures 4E and 4F and Supplementary Figure 13), demonstrating that the increase in *CRABP1* levels contributed to the promotion of cancer progression in the CIC knock-down PC-3 cells.

### *CRABP1* is a direct target gene of CIC in PC-3 cells

**Figure 5:**
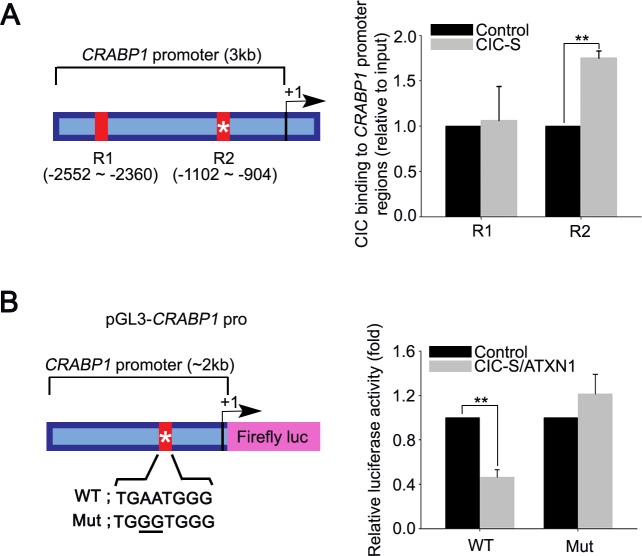
*CRABP1* is a direct target of CIC in PC-3 cells **A.** ChIP-qPCR analyses for CIC occupancy of *CRABP1* promoter in PC-3 cells. PC-3 cells infected with control or FLAG-CIC-S expressing lentivirus were subjected to ChIP using anti-FLAG antibody followed by qPCR for *CRABP1* promoter regions. The left panel is schematic illustration showing the loci of the putative CIC binding motif (asterisk) and PCR amplified regions (R1 and R2) within 3kb region upstream from the *CRABP1* transcriptional start site. Four independent experiments were performed. **P < 0.01. All error bars show s.e.m. **B.** Luciferase assay showing that CIC represses *CRABP1* promoter activity through the CIC binding motif in *CRABP1* promoter. The left panel is a schematic illustration for the luciferase reporter construct harboring the *CRABP1* promoter region (pGL3-*CRABP1* pro) and mutated sequences of the putative CIC binding motif. Four independent experiments were performed. **P < 0.01. All error bars show s.e.m.

We next set out to determine the molecular basis of *CRABP1* overexpression by CIC deficiency. Given that CIC functions as a transcriptional repressor and that the levels of *CRABP1* are increased by CIC deficiency, we hypothesized that *CRABP1* might be a target gene of CIC. To test this hypothesis, we first searched for CIC binding motifs in *CRABP1* promoter region and found a putative CIC binding motif within 3kb region upstream from the transcriptional start site of *CRABP1* (Supplementary Figure 14). We then examined whether CIC binds to the *CRABP1* promoter region containing the CIC binding motif. To this end, we carried out chromatin immunoprecipitation (ChIP) using anti-FLAG antibody in PC-3 cells expressing FLAG-CIC-S followed by qPCR for *CRABP1* promoter regions. The ChIP-qPCR analyses indicate that DNA fragments corresponding to the *CRABP1* promoter region with the CIC binding motif are significantly enriched in the immunoprecipitates of FLAG-CIC-S (Figure 5A), suggesting that CIC might repress *CRABP1* expression through a direct binding to the promoter region in PC-3 cells. To determine that CIC indeed represses *CRABP1* promoter activity, we generated luciferase expression vector driven by *CRABP1* promoter (pGL3-*CRABP1* pro) and carried out dual luciferase assay using this reporter construct. When CIC-S and ATXN1, the latter of which has been known to enhance the transcriptional repressor activity of CIC [5, 20], were co-expressed in PC-3 cells, luciferase activity was significantly decreased, indicating suppression of *CRABP1* promoter activity by CIC (Figure 5B). Moreover, mutagenesis at the putative CIC binding motif abolished the suppressive effect of CIC on *CRABP1* promoter activity (Figure 5B), demonstrating that CIC represses transcription of *CRABP1* through the CIC binding motif. Taken together, our data indicate that *CRABP1* expression is directly regulated by CIC in PC-3 cells.

### miR-93, miR-106b, and miR-375 cooperatively down-regulate CIC levels

**Figure 6:**
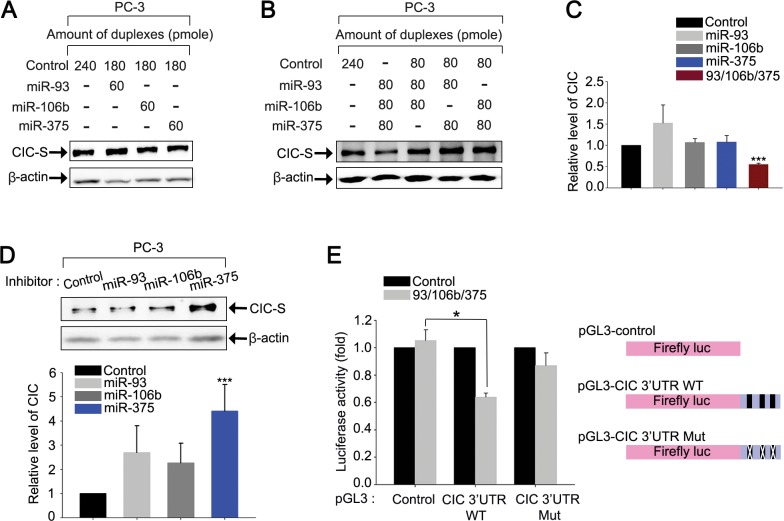
miR-93, miR-106b, and miR-375 cooperatively down-regulate CIC levels in PC-3 cells **A.** Western blot analysis for CIC levels in PC-3 cells transfected with control, miR-93, miR-106b, or miR-375 duplexes. **B.** Western blot analysis for changes in CIC levels by overexpression of miR-93, miR-106b, and miR-375 in PC-3 cells. Co-expression of three miRNAs significantly decreased levels of CIC. **C.** Bar graph for quantitative analysis on the level of CIC based on western blot images. More than three independent experiments were carried out. ***P < 0.001. All error bars show s.e.m. **D.** Western blot analysis for changes in CIC levels by inhibition of endogenous miR-93, miR-106b or miR-375 in PC-3 cells and its quantification. Inhibition of miR-375 significantly increased levels of CIC. More than three independent experiments were carried out. ***P < 0.001. All error bars show s.e.m. **E.** Luciferase assay showing that the 3′UTR of *CIC* is responsible for the miRNAs-mediated down-regulation of CIC expression in PC-3 cells. Three independent experiments were carried out. *P < 0.05. All error bars show s.e.m.

Since CIC levels were markedly decreased in prostate cancer cells, we set out to identify a potential underlying mechanism. It is known that several microRNAs (miRNAs) are abnormally expressed in prostate cancer cells [21, 22], implicating their potential roles in tumorigenesis and/or cancer progression. In this regard, it would be conceivable that overexpression of a subset of miRNAs targeting *CIC* might down-regulate CIC levels to promote cancer progression in the prostate. Thus, we sought to identify miRNAs targeting *CIC* among the miRNAs known to be overexpressed in patients with prostate cancer. Based on previous work [21, 23-27], we searched for miRNAs that have been reported to be up-regulated in samples from prostate cancer patients by at least two independent studies. We also looked for miRNAs potentially targeting *CIC* using miRNA target prediction databases [28, 29]. Comparative analysis on the selected miRNAs identified five miRNAs, miR-20a, miR-25, miR-93, miR-106b, and miR-375, which not only potentially target *CIC*, but are also known to be frequently overexpressed in prostate cancer cells (Supplementary Figure 15A). Since the seed sequences of miR-20a, miR-93, and miR-106b are identical, they are classified as the same miRNA family (miR-17 family). There are two putative binding sites for miR-20a/miR-93/miR-106b, one for miR-25 and another for miR-375 in the 3′UTR of *CIC* (Supplementary Figure 15B). Of the five miRNAs, we initially chose to evaluate miR-93, miR-106b, and miR-375, considering the number of putative binding sites for each miRNA in the 3′UTR of *CIC* and their frequency of overexpression in prostate cancer patients, and tested whether these miRNAs can down-regulate CIC levels. Transfection with each individual miRNA duplex did not significantly affect CIC levels in PC-3 cells (Figures 6A and 6C). However, co-transfection with all three miRNA duplexes markedly down-regulated CIC levels in PC-3 cells (Figures 6B and 6C), indicating that miR-93, miR-106b, and miR-375 cooperatively regulate CIC levels. We did not observe such effect when different combinations of two miRNA duplexes were co-transfected (Figure 6B), suggesting that miR-93 and miR-106b may not function redundantly to regulate CIC levels, although they share the same seed sequences. In fact, among the two putative miR-17 family miRNA binding sites in the *CIC* 3′UTR, the first site is predicted to be more preferentially targeted by miR-93, whereas the second one by miR-106b, according to the miRNA target prediction databases (Supplementary Figure 15B). Moreover, it has been known that nucleotide sequences outside of seed sequences also contribute to determination of target mRNA binding specificity of miRNAs [30, 31]. We also examined whether CIC expression is regulated by endogenous miR-93, miR-106b, and miR-375 in PC-3 cells. Inhibition of miR-375, but not miR-93 and miR-106b, significantly increased CIC levels (Figure 6D), suggesting that, among the three miRNAs, miR-375 is the most critical endogenous miRNA for regulation of CIC levels in PC-3 cells. To verify that miR-93, miR-106b, and miR-375 directly target the 3′UTR of *CIC*, we constructed luciferase reporter gene linked to the *CIC* 3′UTR (pGL3-CIC 3′UTR WT), and carried out dual luciferase assays. Co-transfection with three miRNAs decreased luciferase activity in PC-3 cells (Figure 6E), suggesting that miR-93, miR-106b, and miR-375 down-regulate CIC levels through the 3′UTR of *CIC*. Moreover, disruption of the putative miRNA binding sites in the 3′UTR of *CIC* abrogated suppression of luciferase activity by the three miRNAs (Figure 6E), demonstrating that miR-93, miR-106b, and miR-375 directly target the 3′UTR of *CIC* to regulate CIC levels. On the other hand, the three miRNAs still slightly repressed luciferase activity derived from the pGL3-CIC 3′UTR Mut compared with control vector (Figure 6E), implying that there might be other binding sites for miR-93, miR-106b, and miR-375 in the 3′UTR of *CIC*, or that the three miRNAs might also be able to repress CIC expression indirectly.

### miR-93, miR-106b, and miR-375 cooperatively regulate CIC-CRABP1 axis to promote prostate cancer progression

**Figure 7:**
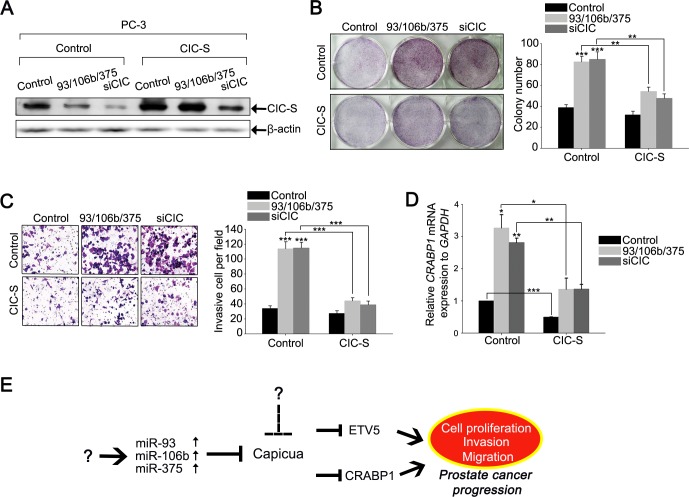
miR-93, miR-106b, and miR-375 co-regulate CIC-CRABP1 axis to promote cancer progression in PC-3 cells **A.** Western blot analysis for CIC levels in PC-3 cells treated with different combinations of three miRNA duplexes or siCIC, and control or CIC-S-expressing lentivirus. **B.** Clonogenic assay for the PC-3 cells used in **A.** and its quantification. Three independent experiments were carried out. **P < 0.01 and ***P < 0.001. All error bars show s.e.m. **C.** Matrigel invasion assay for the PC-3 cells used in **A.** and its quantification. Three independent experiments were carried out. ***P < 0.001. All error bars show s.e.m. **D.** qRT-PCR analysis for *CRABP1* levels in the PC-3 cells used in **A.**. Three independent experiments were carried out. *P < 0.05, **P < 0.01 and ***P < 0.001. All error bars show s.e.m. **E.** The proposed model that describes how prostate cancer progression is regulated by miRNAs, CIC, and its target genes, based on the findings in this study. The question marks mean unidentified factors or mechanisms.

To determine the impact of the miRNAs-mediated down-regulation of CIC on prostate cancer progression, we assessed cell proliferation and invasion in PC-3 cells transfected with either control, miR-93/miR-106b/miR-375 or siCIC duplexes. Overexpression of miR-93, miR-106b, and miR-375 increased cell proliferation and invasion (Figures 7B and 7C and Supplementary Figure 16), accompanied with down-regulation of CIC levels (Figure 7A), suggesting the cancer promoting property of these three miRNAs in prostate cancer cells. To verify that the miRNAs-mediated down-regulation of CIC levels contributed to the enhanced cell proliferation and invasion, we restored CIC levels in PC-3 cells co-transfected with the three miRNA duplexes by mouse CIC-S expressing lentiviral infection (Figure 7A), and conducted clonogenic, cell growth, and invasion assays. The miRNAs-mediated increases in cell proliferation and invasion were partially abolished by recovery of CIC levels in PC-3 cells (Figures 7B and 7C and Supplementary Figure 16), suggesting that miR-93, miR-106b, and miR-375 promote prostate cancer progression in part by down-regulation of CIC expression. We also measured *CRABP1* levels in the same set of cells, and found that co-expression of miR-93, miR-106b, and miR-375 resulted in up-regulation of *CRABP1*, which was restored by overexpression of CIC in PC-3 cells (Figure 7D). Taken together, these data demonstrate that miR-93, miR-106b, and miR-375 function cooperatively to regulate the CIC-CRABP1 axis in promoting prostate cancer progression.

## DISCUSSION

Our study demonstrates that CIC negatively regulates prostate cancer progression and raises a possibility that CIC could be a novel tumor suppressor for prostate cancer. According to The Cancer Genome Atlas (TCGA, http://cancergenome.nih.gov/), the frequencies of deletional and truncating *CIC* gene mutations found in 258 prostate cancer patient samples are 3.1% (8 cases) and 0.4% (1 case), respectively. Examination of how those mutations affect the activity of CIC would be critical to further evaluate the potential role of CIC as a tumor suppressor in the prostate.

In this study, we identified *CRABP1* as a novel CIC target, and provided a molecular basis of how CIC regulates prostate cancer progression. Although a few studies have shown that CRABP1 could promote cancer progression in some types of cancer [18, 32], we now for the first time show that CRABP1 has cancer promoting property in prostate cancer cells. However, it remains unknown how CRABP1 facilitates cell proliferation and invasion in prostate cancer cells. In fact, the binding of retinoic acid, which stimulates differentiation and inhibits proliferation of cells, is the only known function of CRABP1 [33]. On the other hand, it has also been reported that the cancer promoting activity of CRABP1 is independent of the retinoic acid binding activity [18]. Therefore, in-depth molecular characterization of CRABP1 will be required for understanding of the molecular mechanism underlying the promotion of prostate cancer progression by CRABP1.

We found that CIC represses expression of *ETV5* and *CRABP1* in a cell-type dependent manner. This conclusion is further supported by our findings that expression of *ETV5* and *CRABP1* is not up-regulated by knock-down of CIC in DU145 cells whereas levels of *ETV5*, but not *CRABP1*, are significantly up-regulated in CIC knock-down LNCaP-LN3 cells (Supplementary Figure 17). Given that PC-3 cells are highly metastatic and independent of androgen [16], the CIC-CRABP1 axis might play a critical role in regulation of prostate cancer progression at metastatic and castration-resistant stages. The mRNA sequencing analysis also reveals that several genes, such as *NUDT11*, *CCNA1*, and *HNF1B*, known to promote prostate cancer progression [34, 35], are significantly up-regulated in the CIC knock-down PC-3 cells (Supplementary Table 2). In fact, knock-down of CIC more dramatically increased cell proliferation and invasion in PC-3 cells than overexpression of CRABP1 (Figures 2E, 2F and 4C), suggesting that not only *CRABP1*, but other genes regulated by CIC are also involved in promotion of cancer progression by CIC deficiency. Identification of CIC target genes and elucidation of CIC-mediated gene expression regulatory networks at different stages of prostate cancer pathogenesis will uncover CIC-mediated molecular pathways regulating cancer progression in the prostate.

Our data indicate that CIC levels are drastically decreased in prostatic carcinoma. We consistently observed disappearance of the nuclear CIC expression in prostatic adenocarcinoma in all of the tested patient specimens. Moreover, overexpression of CIC markedly suppressed cell proliferation, invasion, and migration in PC-3 cells. Therefore, CIC could be considered as a therapeutic target as well as a molecular marker of prostate cancer. We identified miRNAs targeting *CIC* from the miRNAs known to be overexpressed in prostate cancer tissues, and proposed that miR-93, miR-106b, and miR-375 could potentially contribute to the down-regulation of CIC levels in the process of prostate cancer progression. Comparative miRNA profiling of prostate carcinomas with increasing tumor stages has revealed that levels of miR-375 and miR-106b gradually increase from normal to lymph node metastasizing tumors, whereas miR-93 increases from normal to extracapsular growing tumors [36], suggesting that these miRNAs are likely to participate in the gradual decrease in CIC levels during prostate cancer progression.

In sum, our findings suggest that miR-93/miR-106b/miR-375-CIC-CRABP1 is a novel regulatory axis in prostate cancer progression (Figure 7E). Identification of other components involved in this regulatory axis and further studies on their functions will not only advance the understanding of pathogenesis of prostate cancer, but may also uncover novel therapeutic targets that can be used in the design of treatment modalities for prostate cancer.

## MATERIALS AND METHODS

### Human tissue samples

This study was approved by the Asan Medical Center Institutional Review Board (2011-499). Patients who underwent radical prostatectomy between January 2006 and December 2012 at Asan Medical Center were selected. We randomly chose 13 prostate cancer patients' tissues to detect CIC expression by immunofluorescence staining. According to the value of the Gleason score, the patient's samples were divided into patients with Gleason score with 5-9.

### Immunofluorescence staining

Immunofluorescence staining was performed using standard protocols. Briefly, paraffin sections were dewaxed using xylene for 30 min, and antigen retrieval was performed by boiling in 10mM sodium citrate for 15 min. The sections were treated with rabbit anti-CIC [8] (1:100), chicken anti-Keratin 5 (1:100) (COVANCE, Hertfordshire, England), mouse anti-Cytokeratin 8 (1:500) (COVANCE, Hertfordshire, England), or mouse anti-Cytokeratin 14 (1:100) (BioGenex, CA, USA) antibodies overnight at 4°C, and then incubated with secondary antibodies, Alexa Fluor 488 goat anti-rabbit IgG (Invitrogen, NY, USA), Alexa Fluor 594 goat anti- mouse IgG (Invitrogen, NY, USA) and goat anti-chicken IgY-TR (Santa Cruz Biotechnology, TX, USA) for 1 h at room temperature with 4,6-diamidino-2-phenylindole (DAPI) (Sigma-Aldrich, MO, USA). Immunofluorescence staining was imaged using ZEISS Axioplan2 fluorescence microscope or ZEISS LSM700 confocal microscope.

### Cell culture

PNT2, PC-3, LNCaP, DU145 and LNCaP-LN3 cells were maintained in RPMI 1640 (HyClone, UT, USA) with 10% fetal bovine serum (FBS) and 1% penicillin/streptomycin (WELGENE, Daegu, Republic of Korea). All cell lines were maintained at 37°C in a humid atmosphere containing 5% CO_2_. Instead of trypsin, cell scrapers were used to detach PC-3 cells during subculture of the cells.

### Transfection of siRNA and miRNA duplexes and miRNA inhibitor

miRNA inhibitors, miRNA mimic duplexes, and siRNAs targeting *CRABP1*, *CIC*, and *ETV5* were purchased from Bioneer (Daejun, Republic of Korea). Sequences are as follows: miR-93 sense; 5′-CAAAGUGCUGUUCGUGCAGGUAG-3′, and antisense; 5′-ACCUGCACGAACAGCACUUUAUU-3′. miR-106b sense; 5′-UAAAGUGCUGACAGUGCAGAU-3′, and antisense; 5′-CUGCACUGUCAGCACUUUGUU-3′. miR-375 sense; 5′-UUUGUUCGUUCGGCUCGCGUGA-3′, and antisense; 5′-ACGCGAGCCGAACGAACAAAUU-3′. siETV5 sense; 5′-CACAAGCUUAGAUUCUCUA-3′, and antisense; 5′- UAGAGAAUCUAAGCUUGUG-3′. siCIC sense; 5′-CAGAACGGCUACACACAGU-3′, and antisense; 5′-ACUGUGUGUAGCCGUUCUG-3′. siCRABP1-1 sense; 5′-GUAUCCCUAGUGCUCCAUA-3′, and antisense; 5′-UAUGGAGCACUAGGGAUAC-3′. siCRABP1-2 sense; 5′-CGAAGUCAUUAAACUGGUU-3′, and antisense; 5′-AACCAGUUUAAUGACUUCG-3′. Transfection of siRNA duplexes, miRNA duplexes, or miRNA inhibitors was conducted using Dharmafect 2 (Dharmcon, CO, USA) according to the manufacturer's instructions. The cells were transfected with 120 pmole of siRNA duplexes or 60 pmole of miRNA inhibitors, and incubated for 72∼96 h.

### Clonogenic assay

For clonogenic assay of CIC knock-down PC-3 or LNCaP cells, 2 × 10^3^ or 4 × 10^3^ cells were seeded in six well plates and incubated for 7∼8 days or 14∼15 days, respectively. For clonogenic assay of PC-3 or LNCaP cells overexpressing CIC, 2 × 10^3^ or 4 × 10^3^ cells were seeded in six well plates and incubated for 14∼15 days or 10∼11 days, respectively. For clonogenic assay of PC-3 cells overexpressing FLAG-CRABP1, 2 × 10^3^ cells were seeded in six well plates and incubated for 5 days. The cells were stained with formalin/0.1% crystal violet solution. For clonogenic assay of siRNAs-treated CIC knock-down PC-3 or LNCaP cells, 2 × 10^3^ or 4 × 10^3^ cells were seeded in six well plates a day before transfection, respectively, and then siRNAs were transfected using Dhamafect 2. The cells were stained with formalin/0.1% crystal violet solution after 72 h incubation. For clonogenic assay of PC-3 cells treated with miRNA duplexes and CIC-S expressing lentivirus, 5 × 10^3^ PC-3 cells were seeded in six well plates a day before transfection, and then co-transfected with miR-93, miR-106b, and miR-375 duplexes using Dhamafect 2. After 24 h, the cells were infected with lentivirus expressing CIC-S for 3 sequential days. The cells were stained with formalin/0.1% crystal violet solution and analyzed using Olympus CKX41 microscope and Adobe Photoshop CS6 software. Analyzed cell number is listed in Table S3.

### Cell growth assay

For cell growth assay of PC-3 cells overexpressing FLAG-CRABP1, 1 × 10^3^ cells were seeded in 24 well plates and incubated for 6 days. The cells were trypsinized, stained with Trypan Blue (Sigma-Aldrich, MO, USA), and counted for the number of viable cells using hemacytometer. For cell growth assay of siRNAs-treated CIC knock-down PC-3 or LNCaP cells, 1 × 10^3^ cells were seeded in 24 well plates a day before transfection, and then siRNAs were transfected using Dhamafect 2 and set as day “0”. The cells were trypsinized, stained with Trypan Blue (Sigma-Aldrich, MO, USA), and counted for the number of viable cells using hemacytometer every other day for 6 days. For cell growth assay of PC-3 cells treated with miRNA duplexes and CIC-S expressing lentivirus, 1 × 10^3^ PC-3 cells were seeded in 24 well plates a day before transfection, and then co-transfected with miR-93, miR-106b, and miR-375 duplexes using Dhamafect 2 and set as day “0”. After 24 h, the cells were infected with lentivirus expressing CIC-S for 3 sequential days. The cells were trypsinized, stained with Trypan Blue (Sigma-Aldrich, MO, USA), and counted for the number of viable cells using hemacytometer over 4 days.

### Invasion assay

For invasion assay of CIC knock-down PC-3 or LNCaP cells, 5 × 10^3^ cells were suspended in serum-free medium and seeded into the inserts of a 24-well BioCoat Matrigel Invasion Chamber (BD falcon, CA, USA). For invasion assay of PC-3 or LNCaP cells overexpressing CIC, 1 ×10^4^ or 4 × 10^3^ cells were seeded into the inserts, respectively. For invasion assay of PC-3 cells overexpressing CRABP1, 5 × 10^3^ cells were seeded into the inserts. For invasion assay of siRNAs-treated CIC knock-down PC-3 or LNCaP cells, 2 × 10^3^ or 3 × 10^3^ cells were seeded into the inserts, respectively. For invasion assay of PC-3 cells treated with miRNA duplexes and CIC-S expressing lentivirus, 2 × 10^3^ cells were seeded into the inserts. The inserts were co-cultured in a well of 10% FBS-containing media and incubated for 24h. Inserts were then removed and the upper surface of the membrane was rubbed off to remove non-migrating cells with cotton swabs and washed with PBS. Then, inserts were stained with formalin/0.1% crystal violet solution and analyzed under ZEISS Axioplan2 microscope. Multiple 15∼20 images per insert were acquired, and average counts were calculated. Analyzed cell number is listed in Supplementary Table 3.

### Wound healing assay

For the wound-healing assay, cells were seeded into 6-well plates and cultured at full conﬂuency. A sterile 1ml pipette tip was used to scratch the cells to form a wound. The cells were washed with PBS and cultured in serum-free medium. Wound closure was visualized with ZEISS AxioCamICc1 microscope. Multiple 9 images per well were acquired and average counts were calculated. Analyzed cell number is listed in Supplementary Table 3.

### BrdU labeling assay

For BrdU labeling assay of CIC knock-down PC-3 or LNCaP cells, 2 × 10^3^or 4 × 10^3^cells were seeded in six well plates and incubated for 7∼8 days and 14∼15 days, respectively. For BrdU labeling assay of PC-3 or LNCaP cells overexpressing CIC, 2 × 10^3^ or 6 × 10^3^cells were seeded in six well plates and incubated for 14∼15 days, respectively. BrdU (Sigma-Aldrich, MO, USA) was added in cell culture media to a ﬁnal concentration of 100mM and incubated for 2 h at 37°C with 5% CO_2_. The cells were fixed with 4% paraformaldehyde, incubated with mouse anti-BrdU (1:200) (DSHB, Iowa, USA) overnight at 4°C, and then with Alexa Fluro 594 goat anti-mouse IgG (Invitrogen, NY, USA).

### Cloning

To construct pGL3-CIC 3′UTR, entire human *CIC* 3′UTR sequences (606nt) were amplified from cDNA of MCF7 cells using *Pfu-X* DNA polymerase (SolGent, Daejun, Republic of Korea) and cloned into the pGL3-control vector (Promega, WI, USA). To construct pGL3-*CRABP1* pro, human *CRABP1* promoter region (−1927bp ∼ +2bp) was amplified from MCF7 genomic DNA using *Pfu-X* DNA polymerase and cloned into the pGL3-basic vector (Promega, WI, USA). To make pHAGE-FLAG-CIC-S, and CIC-L, mouse *Cic-S* and *Cic-L* coding sequences were amplified from cDNA of NIH3T3 cells using *Pfu-X* DNA polymerase and cloned into the pHAGE-FLAG lentiviral vector. To make pHAGE-FLAG-CRABP1, human *CRABP1* coding sequences were amplified from cDNA of MCF7 cells using *Pfu-X* DNA polymerase and cloned into the pHAGE-FLAG lentiviral vector.

### Site-directed mutagenesis

Site-directed mutagenesis was performed using QuickChange II XL Site-Directed Mutagenesis kit (Agilent Technologies, CA, USA) according to the manufacturer's instructions. The primers used for mutagenesis at miR-93, miR-106b, and miR-375 binding sites within the *CIC* 3′UTR are as follows: 93-106b mut-1sense;5′-GTGGGGGCTCCTGCGTCTTGCCACAGGCACGGGGAGGGTT-3′, 93-106b mut-1 antisense; 5′- AACCCT CCCCGCCTGTGGCAAGACGCAGGAGCCCCCAC - 3′, 93-106b mut-2 sense;5′GTGACCTTCAGAGCTTTTCGTCTTATGCAAAATGGCTCCT-3′, 93-106b mut-2 antisense; 5‘- AGGAGC CATTTTGCATAAGACGAAAAGCTCTGAAGGTCAC - 3′, 375 mutsense;5′-CTTGCCCCCTTCCCCAGATGTAAACATGTTGATCATGTGC-3′, and 375 mut antisense; TCAACATGTTTACATCTGGGGAAGGGGGCAAG-3′. The primers used for mutagenesis at the CIC binding motif within *CRABP1* promoter are as follows: sense;5′-GGGTTAATCAAATCTTGCCCACCCACGAAAGCCCATCTTTATGC-3′, antisense; 5′- GCATAAAGATGG GCTTTCGTGGGTGGGCAAGATTTGATTAACCC-3′.

### Dual luciferase assay

To examine the effect of miRNAs on the 3′UTR of *CIC*, PC-3 cells seeded in 24-well plates were transfected with pGL3-control, CIC 3′UTR WT or CIC 3′UTR Mut (60ng), together with pRL-TK (15ng), and control or miRNA duplexes using Lipofectamine2000 (Invitrogen, NY, USA) according to the manufacturer's instruction. To examine the effect of CIC and ATXN1 on the *CRABP1* promoter activity, PC-3 cells seeded in 24-well plates were transfected with pGL3-*CRABP1* pro WT or Mut (120ng), together with pRL-TK (30ng), pHAGE-FLAG-CIC-S (60ng) and pcDNA3.1-FLAG-ATXN1 (60ng) [37] using Lipofectamine2000 (Invitrogen, NY, USA) according to the manufacturer's instruction. The cells were lysed 48h later, and luciferase activities were analyzed using the Dual-Luciferase Reporter Assay System (Promega, WI, USA) according to the manufacturer's protocol.

### Lentivirus production and transduction

Non-silencing control shRNA (Cat no. RHS4346) and shCIC expressing lentiviral vectors (pGIPZ) were purchased from Open Biosystems. The clone IDs for shCIC-1, shCIC-2, and shCIC-3 are V3LHS-358902, V3LHS-408353, and V3LHS-358903, respectively. Lentivirus was produced by co-transfection of HEK-293T cells with pGIPZ-shCIC and plasmids for viral particles using FuGENE (Promega, WI, USA). Viral supernatants were collected at 48 h post-transfection and used to infect into the PC-3 and LNCaP cells pre-seeded with approximately 50% confluence. Puromycin was added to select drug-resistant pools at 48 h post-infection. For overexpression of CIC-S, CIC-L, and CRABP1, lentivirus was produced by the same protocol using the cloned pHAGE-FLAG-CIC-S, pHAGE-FLAG-CIC-L, pHAGE-FLAG-CRABP1, or pHAGE-FLAG control plasmid. Viral supernatants were collected at 48 h post-transfection and used to infect into the PC-3 cells for 3 sequential days. The cells were used for further biochemical assays as specified in each experiment.

### Western blot

Cells were lysed with RIPA buffer (50mM Tris (pH 7.4), 150mM NaCl, 0.5% Sodium deoxycholate, 0.1% SDS, 1% Triton X-100) containing protease inhibitor cocktail tablets (Roche, CA, USA). Western blot analysis was performed as described previously [8] using primary antibodies as follows: rabbit anti-CIC (1:1000) [8], rabbit anti-CRABP1 (1:200) (Cell Signaling Technology, MA, USA), mouse anti-β-actin (1:2500) (Santa Cruz Biotechnology, TX, USA), and rabbit anti-FLAG (1:1000) (Sigma-Aldrich, MO, USA). The western blot images were obtained using ImageQuant LAS 4000 (GE Healthcare Life Science, PA, USA) and quantified with Image J software.

### RNA extraction and quantitative RT-PCR

Total RNA was extracted using Trizol reagent (Invitrogen, NY, USA) and subjected to cDNA synthesis using random hexamer and GoScript™ Reverse Transcription System (Promega, WI, USA) according to the manufacturer's instructions. SYBR Green real-time PCR master mix (TOYOBO, NY, USA) was used for PCR reactions. Primers used for qRT-PCR are as follows: *CRABP1* forward; 5′-GCAGCAGCGAGAATTTCGAC-3′, and reverse; 5′-CGTGGTGGATGTCTTGATGTAGA-3′. *ETV1* forward; 5′-ACACCTGTGTTGTCCCAGAA-3′, and reverse; 5′-GTTGGTATGTGGGTCCTTCC-3′. *ETV4* forward; 5′-GATGAAAGCCGGATACTTGGAC-3′, and reverse; 5′-TTCGCGCAAGCTCCCATTT-3′. *ETV5* forward; 5′-CATCCTACATGAGAGGGGGTTA-3′, and reverse; 5′-AAGTATAATCGGGGATCTTTTTCA-3′. *GAPDH* forward; 5′-AGCCACATCGCTCAGACAC-3′, and reverse; 5′-GCCCAATACGACCAAATCC-3′. 18S rRNA forward; 5′-ATCAACTTTCGATGGTAGTCG-3′, and reverse; 5′-ACTCATTCCAATTACAGGGC-3′.

### Chromatin immunoprecipitation

Chromatin immunoprecipitation (ChIP) was performed manually. Briefly, control and FLAG-CIC-S expressing PC-3 cells were fixed in 1% formaldehyde. The cross-linked DNA was then sheared into about 200–1000 base pairs in length with sonication. Ten percent of the sheared DNA was set aside as an input control. The rabbit anti-FLAG antibody (Sigma-Aldrich, MO, USA) was incubated with sheared DNA at 4 °C overnight with rotation, then the protein G agarose (Millipore, Darmstadt, Germany) was added. After that, protein/DNA complexes were eluted from the agarose. The cross-linking DNA was reversed to free DNA, and purified DNA was analyzed by qPCR using the primers that amplify *CRABP1* promoter region containing the CIC-binding site (R2). The primers that amplify *CRABP1* promoter region without CIC-biding sites (R1) were used as a negative control. The primer sequences are as follows: R1 forward; 5′-CAGAGCCAGACCCTGTC-3′, and reverse; 5′-CAGATGAAGGTGTCCACTC-3′. R2 forward; 5′-AAATAATCACAGTTTAGGAAAC-3′, and reverse; 5′-CACCTCAGCCAAACTGTAC-3′.

### RNA sequencing and data analysis

For isolation of mRNA from total RNA, Oligotex mRNA mini kit (QIAGEN, CA, USA) was used according to the manufacturer's instructions. The cDNA was synthesized by random hexamer and Superscript III reverse transcriptase (Invitrogen, NY, USA). The library for the mRNA sequencing was constructed and sequenced on the Genome Analyzer IIx (Illumina, USA) using the protocol described earlier [38]. We mapped sequencing reads to the human reference genome (hg18 RefSeq) using Tophat (v 2.0.9). Reads Per Kilobase of exon per Million aligned tags (RPKM) values for each transcript were calculated by Cufflinks (v 2.2.1) as well as identification of the differential expressed genes. The GEO accession number of RNA sequencing data is GSE64025.

### Statistical analysis

For statistical analysis, all experiments were performed more than three times independently. Data are presented as mean ± standard error. Student's t-test was used to determine significance between groups. For all statistical tests, the 0.05 level of confidence was accepted for statistical significance.

## SUPPLEMENTARY MATERIAL FIGURES AND TABLES



## References

[1] Jiménez G, Guichet A, Ephrussi A, Casanova J (2000). Relief of gene repression by torso RTK signaling: role of capicua in Drosophila terminal and dorsoventral patterning. Genes Dev.

[2] Jiménez G, Shvartsman SY, Paroush Z (2012). The Capicua repressor—a general sensor of RTK signaling in development and disease. J Cell Sci.

[3] Zhu LJ, Christensen RG, Kazemian M, Hull CJ, Enuameh MS, Basciotta MD, Brasefield JA, Zhu C, Asriyan Y, Lapointe DS, Sinha S, Wolfe SA, Brodsky MH (2011). FlyFactorSurvey: a database of Drosophila transcription factor binding specificities determined using the bacterial one-hybrid system. Nucleic Acids Res.

[4] Shin D-H, Hong J-W (2014). Capicua is involved in Dorsal-mediated repression of zerknüllt expression in Drosophila embryo. BMB Rep.

[5] Lam YC, Bowman AB, Jafar-Nejad P, Lim J, Richman R, Fryer JD, Hyun ED, Duvick LA, Orr HT, Botas J, Zoghbi HY (2006). ATAXIN-1 interacts with the repressor Capicua in its native complex to cause SCA1 neuropathology. Cell.

[6] Fryer JD, Yu P, Kang H, Mandel-Brehm C, Carter AN, Crespo-Barreto J, Gao Y, Flora A, Shaw C, Orr HT, Zoghbi HY (2011). Exercise and genetic rescue of SCA1 via the transcriptional repressor Capicua. Science.

[7] Lee Y, Fryer JD, Kang H, Crespo-Barreto J, Bowman AB, Gao Y, Kahle JJ, Hong JS, Kheradmand F, Orr HT, Finegold MJ, Zoghbi HY (2011). ATXN1 protein family and CIC regulate extracellular matrix remodeling and lung alveolarization. Dev Cell.

[8] Kim E, Park S, Choi N, Lee J, Yoe J, Kim S, Jung HY, Kim KT, Kang H, Fryer JD, Zoghbi HY, Hwang D, Lee Y (2015). Deficiency of Capicua disrupts bile acid homeostasis. Sci Rep.

[9] Sjöblom T, Jones S, Wood LD, Parsons DW, Lin J, Barber TD, Mandelker D, Leary RJ, Ptak J, Silliman N, Szabo S, Buckhaults P, Farrell C (2006). The consensus coding sequences of human breast and colorectal cancers. Science.

[10] Kan Z, Jaiswal BS, Stinson J, Janakiraman V, Bhatt D, Stern HM, Yue P, Haverty PM, Bourgon R, Zheng J, Moorhead M, Chaudhuri S, Tomsho LP (2010). Diverse somatic mutation patterns and pathway alterations in human cancers. Nature.

[11] Alentorn A, Sanson M, Idbaih A (2012). Oligodendrogliomas: new insights from the genetics and perspectives. Curr Opin Oncol.

[12] Kawamura-Saito M, Yamazaki Y, Kaneko K, Kawaguchi N, Kanda H, Mukai H, Gotoh T, Motoi T, Fukayama M, Aburatani H, Takizawa T, Nakamura T (2006). Fusion between CIC and DUX4 up-regulates PEA3 family genes in Ewing-like sarcomas with t(4;19)(q35;q13) translocation. Hum Mol Genet.

[13] Kurpios NA, Sabolic NA, Shepherd TG, Fidalgo GM, Hassell JA (2003). Function of PEA3 Ets transcription factors in mammary gland development and oncogenesis. J Mammary Gland Biol Neoplasia.

[14] Dissanayake K, Toth R, Blakey J, Olsson O, Campbell DG, Prescott AR, MacKintosh C (2011). ERK/p90(RSK)/14-3-3 signalling has an impact on expression of PEA3 Ets transcription factors via the transcriptional repressor capicúa. Biochem J.

[15] Clark JP, Cooper CS (2009). ETS gene fusions in prostate cancer. Nat Rev Urol.

[16] Russell PJ, Kingsley EA (2003). Human prostate cancer cell lines. Methods Mol Med.

[17] Oh S, Shin S, Janknecht R (2012). ETV1, 4 and 5: an oncogenic subfamily of ETS transcription factors. Biochim Biophys Acta.

[18] Kainov Y, Favorskaya I, Delektorskaya V, Chemeris G, Komelkov A, Zhuravskaya A, Trukhanova L, Zueva E, Tavitian B, Dyakova N, Zborovskaya I, Tchevkina E (2014). CRABP1 provides high malignancy of transformed mesenchymal cells and contributes to the pathogenesis of mesenchymal and neuroendocrine tumors. Cell Cycle.

[19] Sirotnak FM, She Y, Khokhar NZ, Hayes P, Gerald W, Scher HI (2004). Microarray analysis of prostate cancer progression to reduced androgen dependence: studies in unique models contrasts early and late molecular events. Mol Carcinog.

[20] Crespo-Barreto J, Fryer JD, Shaw CA, Orr HT, Zoghbi HY (2010). Partial loss of ataxin-1 function contributes to transcriptional dysregulation in spinocerebellar ataxia type 1 pathogenesis. PLoS Genet.

[21] Ambs S, Prueitt RL, Yi M, Hudson RS, Howe TM, Petrocca F, Wallace TA, Liu CG, Volinia S, Calin GA, Yfantis HG, Stephens RM, Croce CM (2008). Genomic profiling of microRNA and messenger RNA reveals deregulated microRNA expression in prostate cancer. Cancer Res.

[22] Ozen M, Creighton CJ, Ozdemir M, Ittmann M (2008). Widespread deregulation of microRNA expression in human prostate cancer. Oncogene.

[23] Porkka KP, Pfeiffer MJ, Waltering KK, Vessella RL, Tammela TLJ, Visakorpi T (2007). MicroRNA expression profiling in prostate cancer. Cancer Res.

[24] Tong AW, Fulgham P, Jay C, Chen P, Khalil I, Liu S, Senzer N, Eklund AC, Han J, Nemunaitis J (2009). MicroRNA profile analysis of human prostate cancers. Cancer Gene Ther.

[25] Schaefer A, Jung M, Mollenkopf H-J, Wagner I, Stephan C, Jentzmik F, Miller K, Lein M, Kristiansen G, Jung K (2010). Diagnostic and prognostic implications of microRNA profiling in prostate carcinoma. Int J Cancer.

[26] Wach S, Nolte E, Szczyrba J, Stöhr R, Hartmann A, Ørntoft T, Dyrskjøt L, Eltze E, Wieland W, Keck B, Ekici AB, Grässer F, Wullich B (2012). MicroRNA profiles of prostate carcinoma detected by multiplatform microRNA screening. Int J Cancer.

[27] Szczyrba J, Löprich E, Wach S, Jung V, Unteregger G, Barth S, Grobholz R, Wieland W, Stöhr R, Hartmann A, Wullich B, Grässer F (2010). The microRNA profile of prostate carcinoma obtained by deep sequencing. Mol Cancer Res.

[28] Lewis BP, Burge CB, Bartel DP (2005). Conserved seed pairing, often flanked by adenosines, indicates that thousands of human genes are microRNA targets. Cell.

[29] Krek A, Grün D, Poy MN, Wolf R, Rosenberg L, Epstein EJ, MacMenamin P, da Piedade I, Gunsalus KC, Stoffel M, Rajewsky N (2005). Combinatorial microRNA target predictions. Nat Genet.

[30] Grimson A, Farh KK-H, Johnston WK, Garrett-Engele P, Lim LP, Bartel DP (2007). MicroRNA targeting specificity in mammals: determinants beyond seed pairing. Mol Cell.

[31] Shin C, Nam J-W, Farh KK-H, Chiang HR, Shkumatava A, Bartel DP (2010). Expanding the microRNA targeting code: functional sites with centered pairing. Mol Cell.

[32] Won JY, Nam E-C, Yoo SJ, Kwon HJ, Um SJ, Han HS, Kim SH, Byun Y, Kim SY (2004). The effect of cellular retinoic acid binding protein-I expression on the CYP26-mediated catabolism of all-trans retinoic acid and cell proliferation in head and neck squamous cell carcinoma. Metab Clin Exp.

[33] Dong D, Ruuska SE, Levinthal DJ, Noy N (1999). Distinct roles for cellular retinoic acid-binding proteins I and II in regulating signaling by retinoic acid. J Biol Chem.

[34] Wegiel B, Bjartell A, Tuomela J, Dizeyi N, Tinzl M, Helczynski L, Nilsson E, Otterbein LE, Härkönen P, Persson JL (2008). Multiple cellular mechanisms related to cyclin A1 in prostate cancer invasion and metastasis. J Natl Cancer Inst.

[35] Grisanzio C, Werner L, Takeda D, Awoyemi BC, Pomerantz MM, Yamada H, Sooriakumaran P, Robinson BD, Leung R, Schinzel AC, Mills I, Ross-Adams H, Neal DE (2012). Genetic and functional analyses implicate the NUDT11, HNF1B, and SLC22A3 genes in prostate cancer pathogenesis. Proc Natl Acad Sci USA.

[36] Hart M, Nolte E, Wach S, Szczyrba J, Taubert H, Rau TT, Hartmann A, Grässer FA, Wullich B (2014). Comparative microRNA profiling of prostate carcinomas with increasing tumor stage by deep sequencing. Mol Cancer Res.

[37] Lee Y, Samaco RC, Gatchel JR, Thaller C, Orr HT, Zoghbi HY (2008). miR-19, miR-101 and miR-130 co-regulate ATXN1 levels to potentially modulate SCA1 pathogenesis. Nat Neurosci.

[38] Choe MK, Hong C-P, Park J, Seo SH, Roh T-Y (2012). Functional elements demarcated by histone modifications in breast cancer cells. Biochem Biophys Res Commun.

