# Supplementary material for: miR-93/miR-106b/miR-375-CIC-CRABP1: a novel regulatory axis in prostate cancer progression
Source: Oncotarget. 2015 Jun 8;6(27):23533–47. doi: 10.18632/oncotarget.4372 (PMC4695135; doi:10.18632/oncotarget.4372)
Supplement: Supplementary file 1 [file oncotarget-06-23533-s001.pdf]

miR-93/miR-106b/miR-375-CIC-CRABP1: a novel regulatory axis in prostate cancer progression

Supplementary Material

Figure S1. Patterns of CIC expression in normal mouse prostate tissues.
Figure S2. Test of rabbit polyclonal anti-CIC antibody for immunofluorescence staining.
Figure S3. Comparison of CIC levels in prostate cell lines.
Figure S4. Decreased cell proliferation in PC-3 and LNCaP cells by CIC overexpression.
Figure S5. Changes in cell migration in PC-3 cells by overexpression or knock-down of CIC.
Figure S6. Increased cell proliferation in CIC knock-down PC-3 and LNCaP cells.
Figure S7. Comparable expression of *PEA3* group genes among control and CIC knock-down PC-3 cells.
Figure S8. Derepression of *ETV5* contributes to the increased cell proliferation in CIC knock-down LNCaP cells.
Figure S9. Comparison of *CRABP1* levels in PNT2, LNCaP, and PC-3 cells.
Figure S10. Up-regulation of *CRABP1* expression in PC-3 cells by CIC deficiency.
Figure S11. Comparable expression of *CRABP1* among control and CIC knock-down LNCaP cells.
Figure S12. Overexpression of CRABP1 promotes cell proliferation in PC-3 cells.
Figure S13. Overexpression of *CRABP1* contributes to the increased cell proliferation in CIC knock-down PC-3 cell lines.
Figure S14. CIC binding motif in human *CRABP1* promoter region.
Figure S15. Schematic illustration showing selection of miRNA candidates with potential to target CIC from the miRNAs overexpressed in prostate cancer patients and their binding sites in the 3'UTR of CIC.
Figure S16. The miRNAs-mediated down-regulation of CIC levels contributes to the increase in cell proliferation in PC-3 cells.
Figure S17. Effect of CIC knock-down on expression of *ETV5* and *CRABP1* in DU145 and LNCaP-LN3 cells.

Supplementary Tables

Table S1. The number of non-cancerous prostate gland, PIN, and prostatic adenocarcinoma regions found in each section of 13 prostate cancer patient samples and the percentage of CIC^{+} cells in prostate glands for each phenotype.
Table S2. Differentially expressed genes in CIC knock-down PC-3 cells.
Table S3. Raw data for clonogenic, invasion, BrdU labeling, and wound healing assays.

Supplementary Figure 1

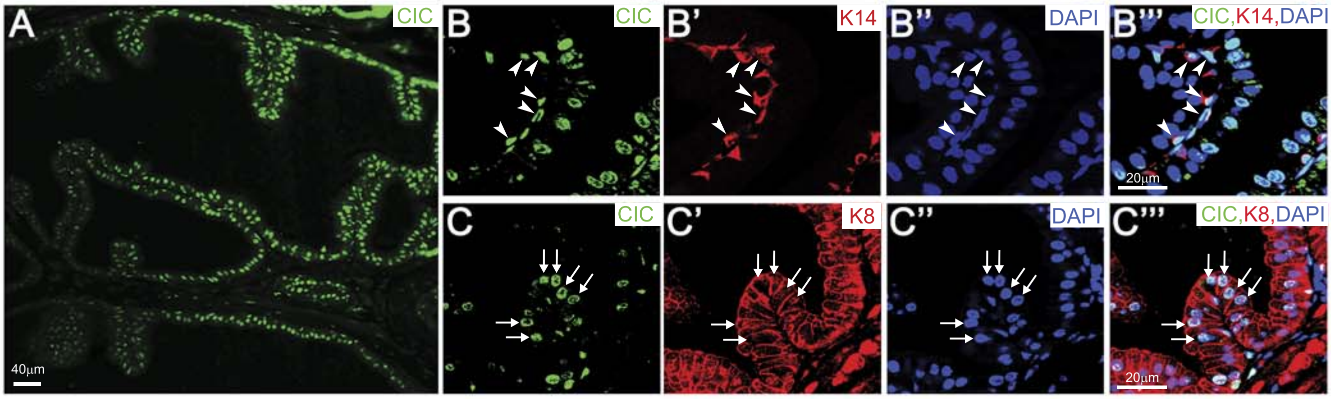

Supplementary Figure 1. Patterns of CIC expression in normal mouse prostate tissues. Immunofluorescence staining showing expression of CIC in the nuclei of basal and luminal cells of mouse prostate glands. The prostate tissues were prepared from 2 month old C57BL/6 male mice.

(A) Low magnification image for expression of CIC in prostate cells.

(B~B''') Images showing nuclear expression of CIC in basal cells (arrowhead). Keratin 14 (K14) is a marker protein for basal cells of prostate glands.

(C~C''') Images showing nuclear expression of CIC in luminal cells (arrow). Keratin 8 (K8) is a marker protein for luminal cells of prostate glands.

Supplementay Figure 2

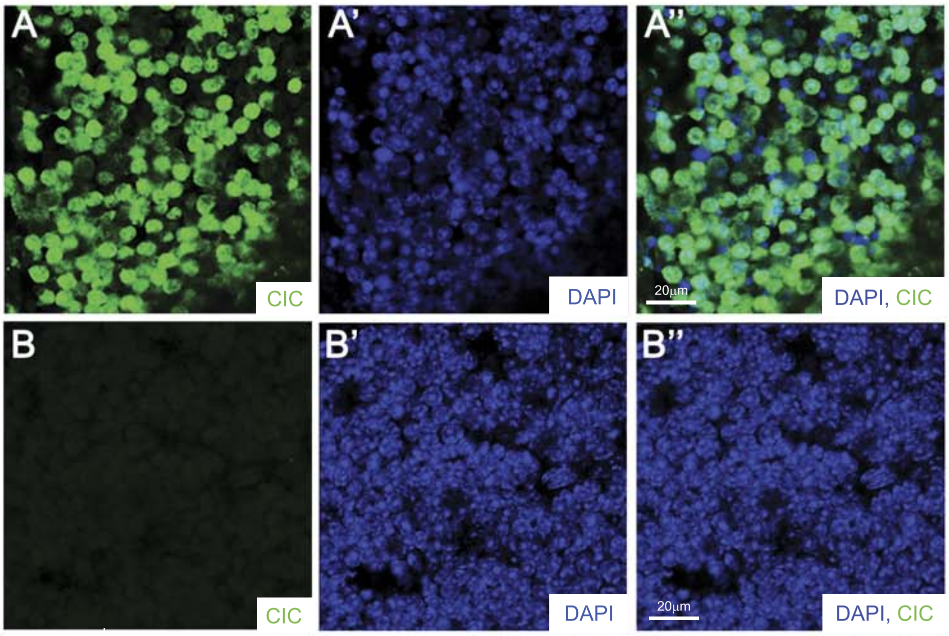

Supplementary Figure 2. Test of rabbit polyclonal anti-CIC antibody for immunofluorescence staining.

Thymus tissues were prepared from WT **(A~A'')** and Cic-L KO **(B~B'')** mice at P18, and subjected to immunofluorescence staining using rabbit polyclonal anti-CIC antibody (green). Green fluorescence signal was markedly reduced in Cic-L KO thymus compared with WT, indicating that the anti-CIC antibody specifically recognizes CIC.

Supplementary Figure 3

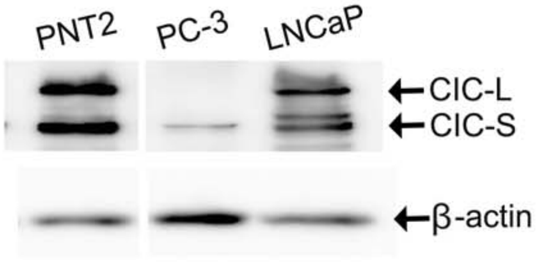

Supplementary Figure 3. Comparison of CIC levels in prostate cell lines. Western blot analysis for CIC levels in PNT2, PC-3, and LNCaP cells. Twenty five μg of cell lysate were loaded on each lane.

Supplementary Figure 4

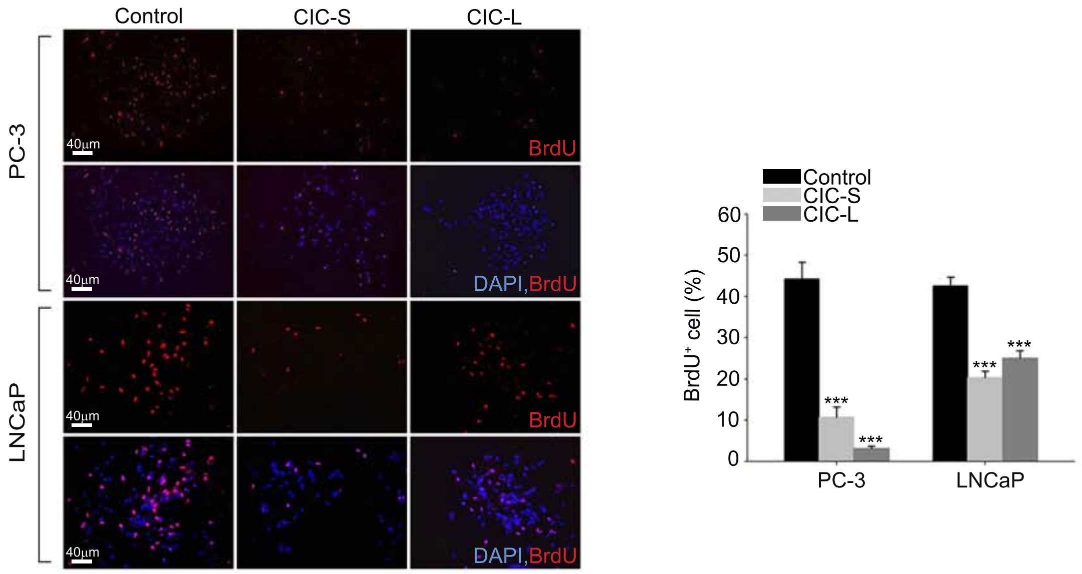

Supplementary Figure 4. Decreased cell proliferation in PC-3 and LNCaP cells by CIC overexpression. BrdU labeling assay showing suppression of cell proliferation by CIC overexpression in PC-3 and LNCaP cells and its quantification. ***P<0.001. All error bars show s.e.m.

Supplementary Figure 5

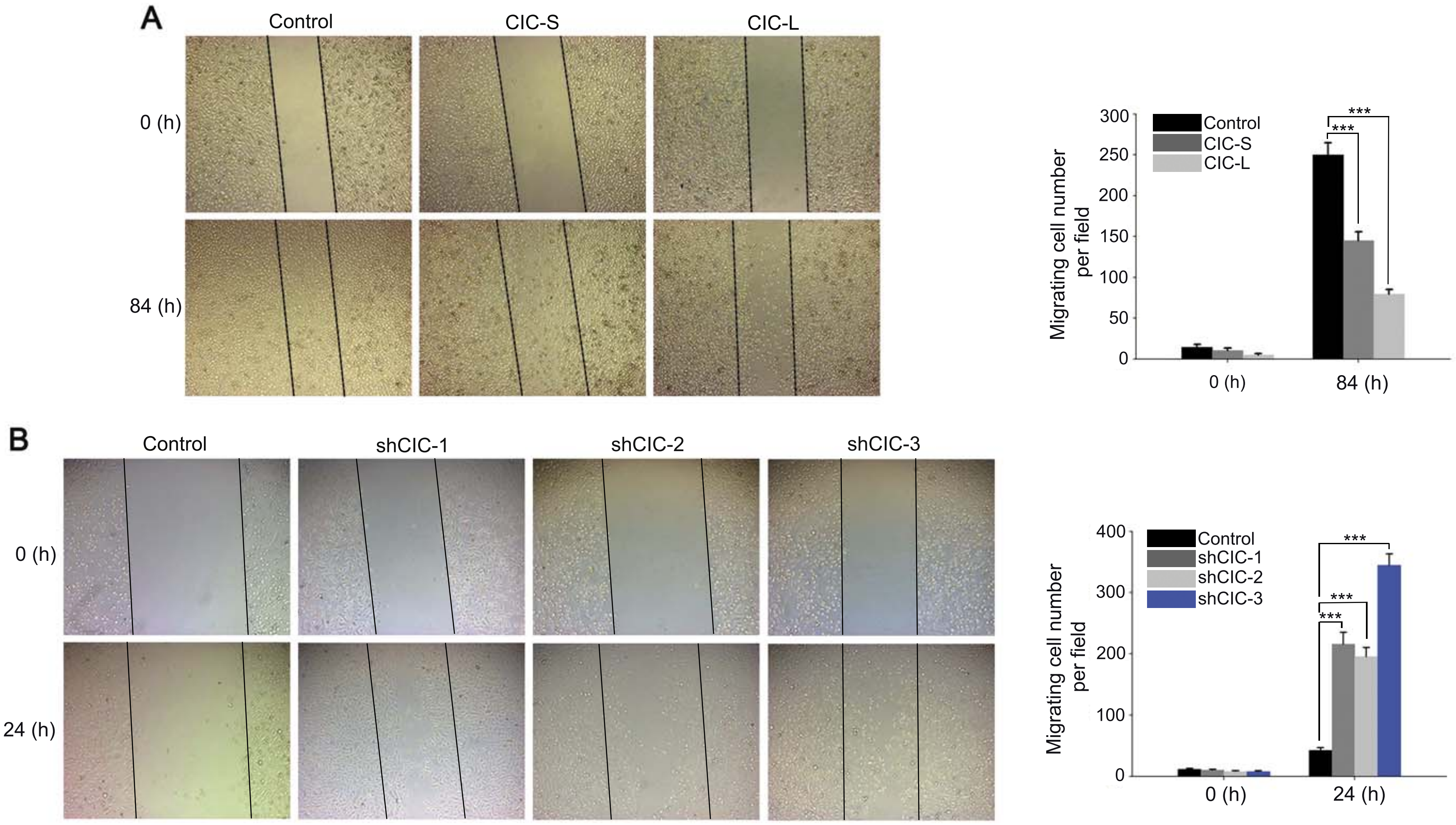

Supplementary Figure 5. Changes in cell migration in PC-3 cells by overexpression or knock-down of CIC.
(A) Wound healing assay showing suppression of cell migration by CIC overexpression in PC-3 cells. The right panel is a bar graph for quantitative analysis on migrating cell numbers. Three independent experiments were performed. ***P<0.001. All error bars show s.e.m.
(B) Wound healing assay showing increased cell migration by knock-down of CIC in PC-3 cells and its quantification. Three independent experiments were performed. ***P<0.001. All error bars show s.e.m.

Supplementary Figure 6

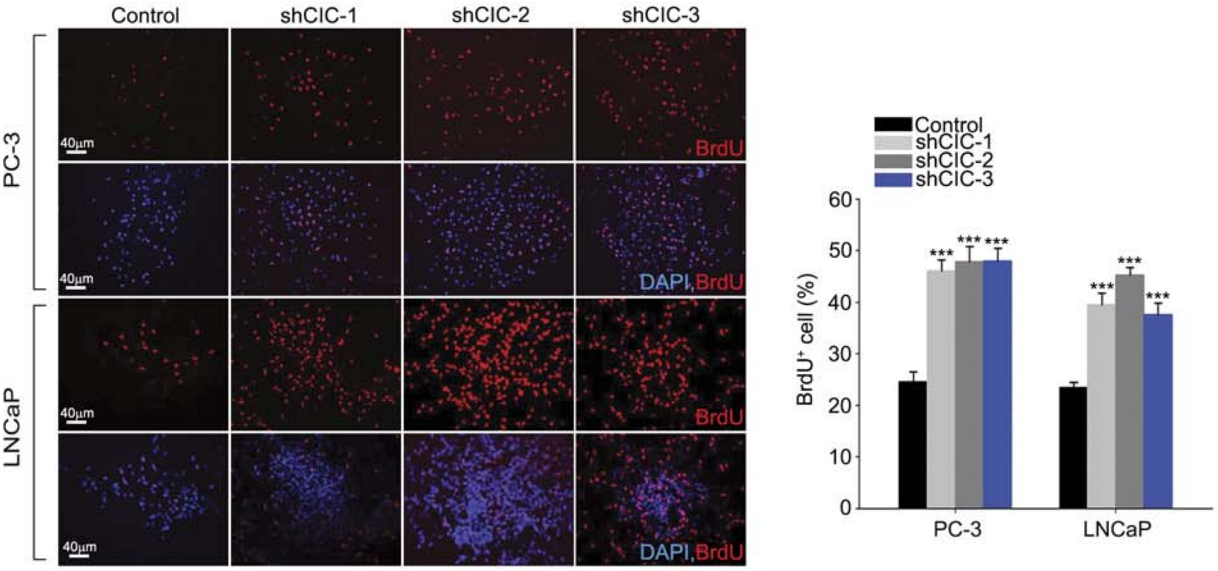

Supplementary Figure 6. Increased cell proliferation in CIC knock-down PC-3 and LNCaP cells. BrdU incorporation assay showing promotion of cell proliferation by knock-down of CIC in PC-3 and LNCaP cells and its quantification. ***P<0.001. All error bars show s.e.m.

Supplementary Figure 7

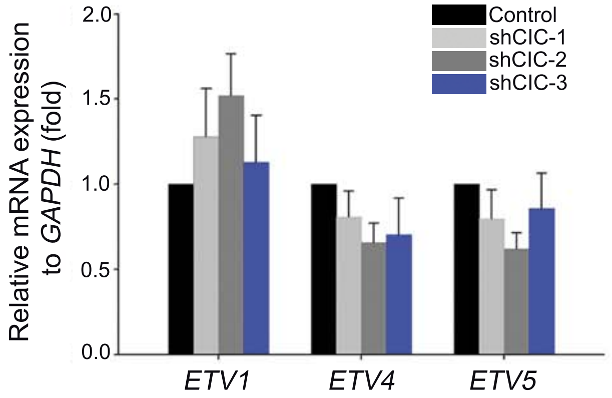

Supplementary Figure 7. Comparable expression of *PEA3* group genes among control and CIC knock-down PC-3 cells.
qRT-PCR analysis for levels of *ETV1*, *ETV4*, and *ETV5* in CIC knock-down PC-3 cells. Three independent experiments were performed. All error bars show s.e.m.

Supplementary Figure 8

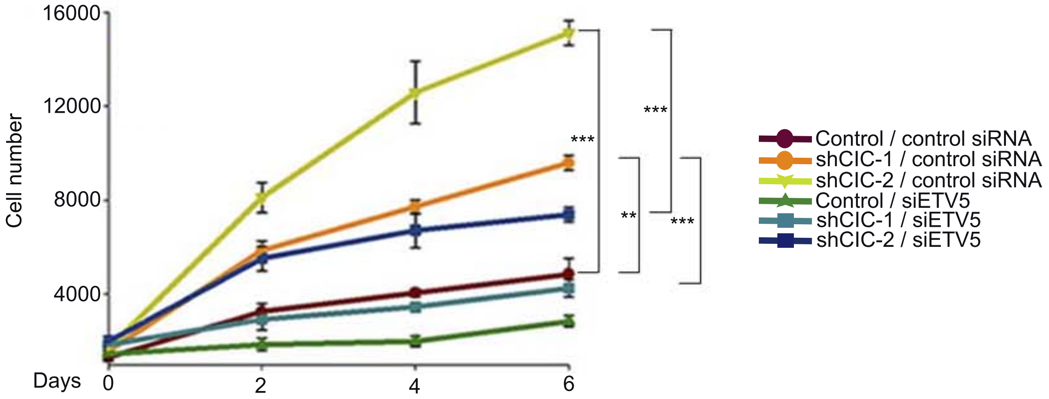

Supplementary Figure 8. Derepression of *ETV5* contributes to the increased cell proliferation in CIC knock-down LNCaP cells.

Cell growth assay for control, shCIC-1 and shCIC-2 LNCaP cells treated with either control or ETV5 siRNA. The number of cells was counted every other day for 6 days using hemacytometer. Three independent experiments were performed. **P<0.01 and **P<0.001. All error bars show s.e.m.

Supplementary Figure 9

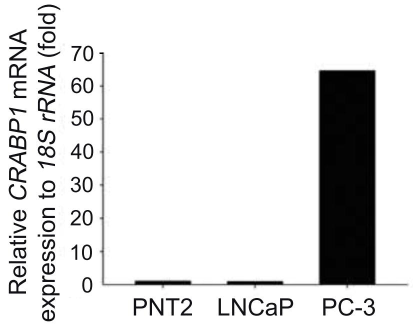

Supplementary Figure 9. Comparison of *CRABP1* levels in PNT2, LNCaP, and PC-3 cells. qRT-PCR analysis for levels of *CRABP1* mRNA in PNT2, LNCaP, and PC-3 cells.

Supplementary Figure 10

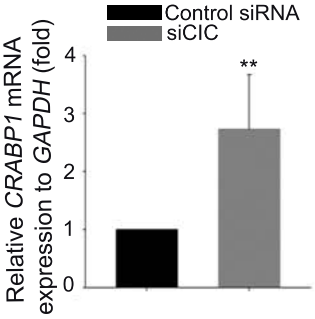

Supplementary Figure 10. Up-regulation of *CRABP1* expression in PC-3 cells by CIC deficiency. qRT-PCR analysis for *CRABP1* levels in PC-3 cells transiently transfected with CIC siRNA duplexes. Five independent experiments were performed. **P<0.01. All error bars show s.e.m.

Supplementary Figure 11

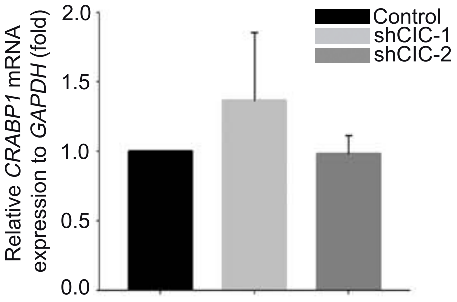

Supplementary Figure 11. Comparable expression of *CRABP1* among control and CIC knock-down LNCaP cells.
qRT-PCR analysis for *CRABP1* levels in control and CIC-knock-down LNCaP cells. Three independent experiments were performed. All error bars show s.e.m.

Supplementary Figure 12

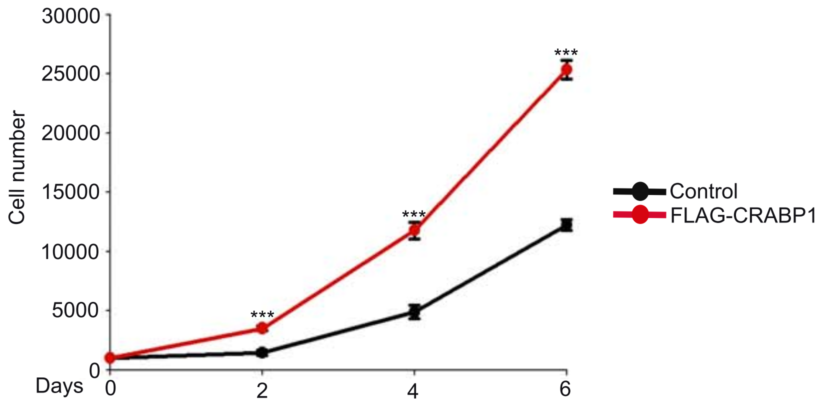

Supplementary Figure 12. Overexpression of CRABP1 promotes cell proliferation in PC-3 cells.
Cell growth assay for control and CRABP1 overexpressed PC-3 cells. The number of cells was counted every other day for 6 days using hemacytometer. Six experiments were performed independently. ***P<0.001. All error bars show s.e.m.

Supplementary Figure 13

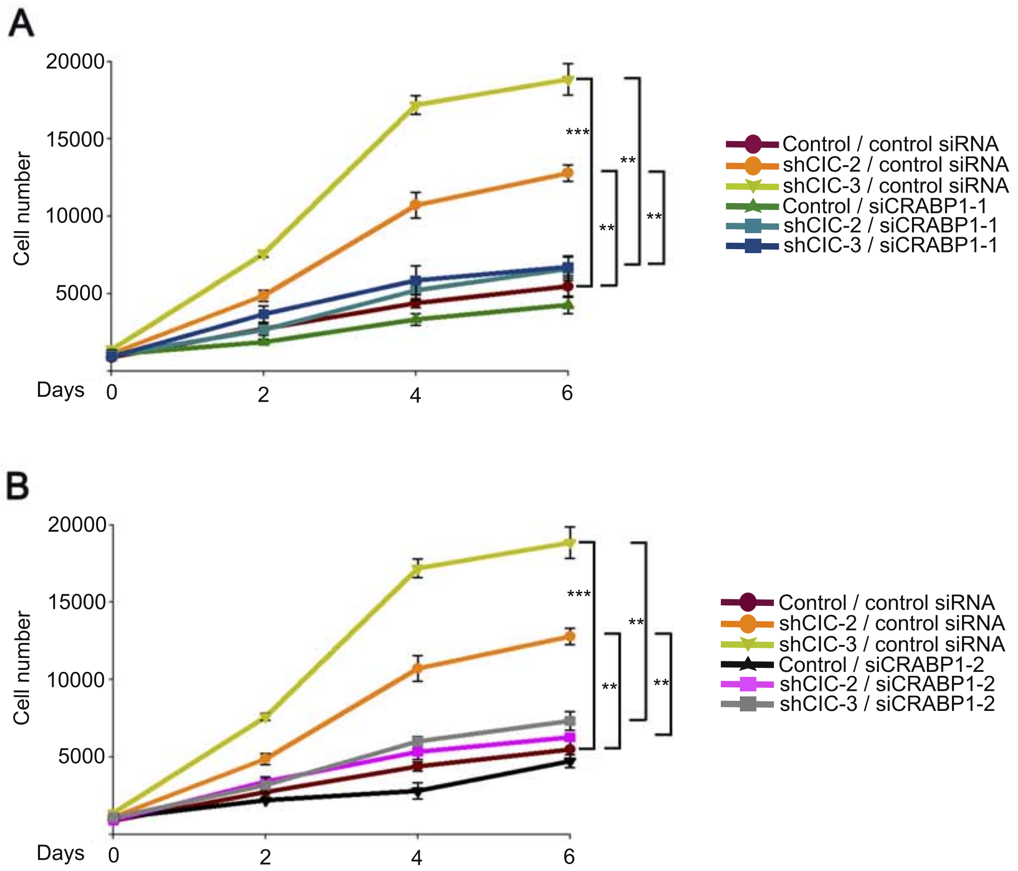

Supplementary Figure 13. Overexpression of *CRABP1* contributes to the increased cell proliferation in CIC knock-down PC-3 cell lines.

Cell growth assay for control and CIC knock-down PC-3 cell lines treated with control or two different CRABP1siRNAs: (**A**) siCRABP1-1 and (**B**) siCRABP1-2. The number of cells was counted every other day for 6 days using hemacytometer. Three experiments were performed independently. **P<0.01 and ***P<0.001. All error bars show s.e.m.

Supplementary Figure 14

CACCGTGCCCAGCCATGGTGGTGGTGTTTTTGTGGCCCTCTTGACTCTGGGGTTTCCCCAGTAGC
CAGGAAGGTGATAGCAGCCTGCCCTCCCATTTCCATGTGCCACCTGATGAGATTTGGGTCTTCTG
AGAAGTCCTGGCCCCTCCTGTCTGCTCTGGTCACCTCTAAACTTCCATTAACAAGGACAGCAGGC
TGGGTGCTCATGCCTGTAATCCTAGCACTTTGGGAGGCTGAGGTGGACAAACTGGTTGAGCTCAG
GAGTTCGAGACCATCCTGGGCAACATGGCAAACCCCTGTCTCTACTAAAAATACAAAAATTAGCC
AGGCACGGTGGTGCATGCCTGTAATCCCAGCTACTTGGGAGGCTGAGGCAGGAGGATTGCTTGAG
CCCGGGAGGCAAGGTTACAGTGACCCAAGATGGCACCAGGGCACTCCAGCACTGGCGACAGAGCC
AGACCCTGTCTTAAAAACAAGGAGAGTGGGCTCACAAGGACTCTGGGATCATCTTCCACTCTATG
GCTGAACTGGCTGTGCCCTCCTCACTCCTGGACCCTGCTCCCGCCCTTGTTTTGTGCCTGCAGAA
CGACACCACTGTCCTGTCCCAGTATGGCCAACTTTTGGAGTGGACACCTTCATCTGGCCCCTCTT
GCCCCATTCTCTCTCTGACTCCCTCCCTCACCTTCTTCTTTCCCTCTTCCCTCTTTGTCCATCCA
ATGCCTTTGCTGCAAGAGCTCTCTGTCACCTTTGTGCCTCATCCACGGCCCTCTGACTATCCTCA
CAAGGTCTCTCTGGGCTGTAAACTAGGATGCTGCTCCAACATTGCTGCCCTCCTCCAGCCTCCTG
CCCCAGTCCCTCCCCAGCCCTGCCCTTTAGATGGCTGAAGCCTTCTTAGGGAAGAGTGTTGGATG
GGGGCAGAGGCCTGCATTAGCTGGATGGCACTCCTGTTTTGCCCCTTCACTGGAGAGGCACTCAA
CCCTCTGTGAAGTGGGTTTGATTCTATGGCAGCTCCATCCCTTGCCTCCCACATCCAGGACCTGC
TACTTAATTTGCAGATCCCAGTGCAAAATAAAAATTCAGGACTCCTTGTTCAAAAAGTATTATGG
GGCCAGGCACGGTGGCTCATGCCTGTAATCCCAGCACTTTGGGAGGCCGAGGAGGGCGGATCACG
AGGTCAGGAGATCGAGACTATCCTGGCTAACACAGTGAAACCCTGTCTCTACTAAAAATACAAAA
AATTAGCTGGGCATAGTAGCACACACCTGTAGTCCCAGCTACTTGGGAGGCTGAGCCAGGAGAAT
CATTTAAACCCAGGAGGCAGAGGTTGCAGTGAGCCAAGATTGCGCCACTGCACTCCAGCTCGGGC
AACAGAGCGAGACTCTGTCTCAAAACAAACAAACAAACAAAAAGTATTCTGACGTTCAAGCTGGC
AATAGCAGAGCATTCCTGCTTTTCCATTTTCCAATCTAACTATCAGGCCCTTCTAAGTGCATGGC
CCTGTGTGACTGCACAGACGGCACGTCCAGAATGCCGGCCCTGCCCATGCCCCTTTCCAGCTGGT
GATGGCCCCACTCCCCTCTCCTGTCCTCAGCTGCTTTCTTAGACAGATCCTCGGAGCCTCCTGGC
AGCAACCCAGCCCCAGCCACCTCCAGAAGTTTCCAGGGCCCTGTTCAGTAGGCACTCAGCACAGA
TTCCCCAAGGTCCTGGGGGAATCTTCACCCCAGGCCACCACTCACATCCTCCTCAAATCAATGCA
CAGTTATCGAGCCCCACCTGGCTAAGCCTTAGGAAGGGAGGACAAGTGGATAAACGATCACTGCC
CTCCAGGAGTTCAGTTTATAGCAGAGCGAAGACTTGAACAAGAGCAGGACCGGACAAGAATCCTG
TCAATTCAGAGAAAAATAATCACAGTTTATTAGGAAACTGAATTTCACAAAAGAGAGAGCATAAA
GATGGGCTTTCG**TGAATGGG**CAAGATTTGATTAACCCGATGAGGAAAGGGGCATTCAAGGAAGAA
GCCATGGCATGGGCAAAAACTTAGAGATAATAAAATGCATGGCACGTTCTAGAATCCTGAATTGT
ACAGTTTGGCTGAGGTGTGCAGATGGTAAAATAGTGGAAAAAGCTGGAAGGAAAATGTACAGAG
AAGAGCTACAAGGGTAATAGTGAAGTGTTTGTACTCCCTTAGTGGAAACACCGAGGGGACTTTAG
GTCGTGATGTTTAGGTTATTTGGAGCAAAGGGAGAAGACTTCCTCCCCTTGGCCCTCACAGCCT
GCCCGAAGAGTGCAAAGCCTCACGCTTTTGCAAAACAGGCCGCACCTAGCCCAGATTCTGGAGCT
GATTCAGTCCATGCCTCTCGACCCACAGTTTGTCCTGAGCCCTGACAAAGGTCTGGCCCCCTCCT
GCACTCGGAACGCGGAGGACCCGCAGCTCGTGGATTCGCGCCTCAGTGCCCAAGCTGGTGTTGAT
GGGGGTTGCGGTTTCCTGCCACTCACTCCCCATGGGTCCTGTGGGGGGCGTGGGGATGCGGGGAG
AGGGTTTCAGAATAGGATCCCGGACTGGGAGGGAAGGGACACCATCTGGGCACACCCAGTAGGTA
CGACCGGAGGTGAAAAAAGAGTGGGTCCCATCCCCTGATGGCCCCGCTCGCTATCCACTTTAAGT
TTATGTTATGGATATTTTCAAACATATTCAAAAGTAGAGAGAAAAGTAGAATCATAACCCAAGTC
CACACCGCCCAGCTTCAACAGTTATCCCCTCCAAGCCCATCTTGTTTCAGCTAACCCTCTCCCAC
CACCCATTTTAAAGCGGGATGTGGGTGTCCTGAGGGAGGTGGAGGCTCCCCAGTTGGAGAGCGGG
TGCCCGCACCTCTGGAGGCTGAGGCACAACTGGGCTGGGAGTTGAGCGCATACCGAGTAGGGGCT
GCGGGGGAGCGGGGGCAGGGCCGGCGGGGCGGGGCCAGAAGCCCGGCGGGGTTGGGGGCGCTCTC
CTAGTGACTC

Supplementary Figure 14. CIC binding motif in human *CRABP1* promoter region.
DNA sequences of *CRABP1* promoter region (3kb upstream from the transcriptional start site of *CRABP1*). One putative CIC binding motif is underlined.

Supplementary Figure 15

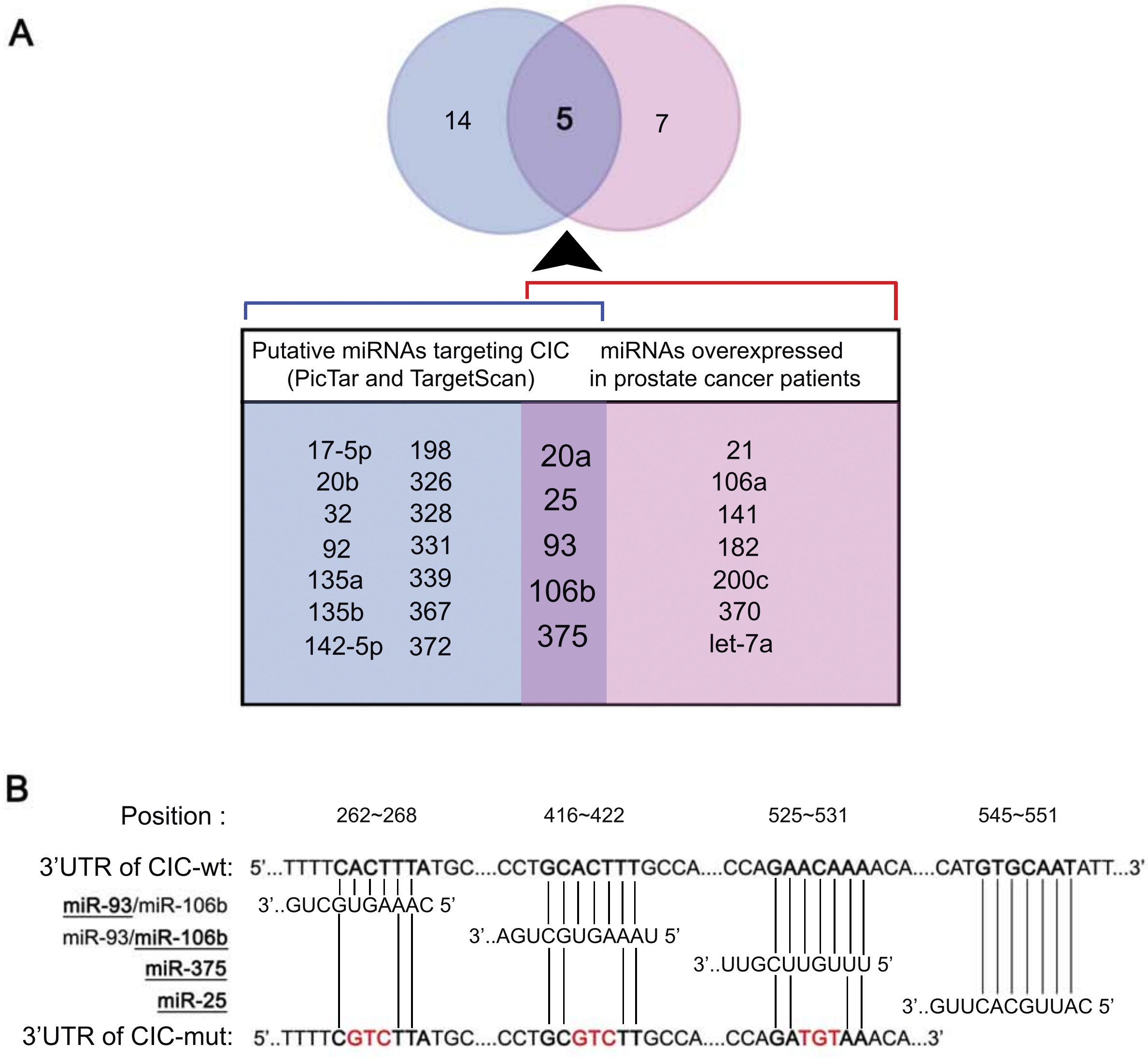

Supplementary Figure 15. Schematic illustration showing selection of miRNA candidates with potential to target *CIC* from the miRNAs overexpressed in prostate cancer patients and their binding sites in the 3'UTR of *CIC*.

(A) Schematic diagram for how the candidate miRNAs were selected. PicTar and TargetScan were used for selection of putative miRNAs targeting *CIC*.

(B) Schematic diagram showing location of putative binding sites for the selected miRNAs in the 3'UTR of *CIC* and mutated sequences.

Supplementary Figure 16

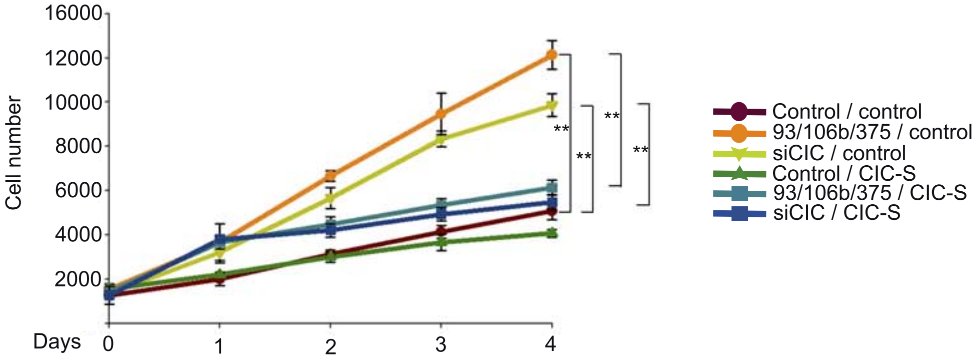

Supplementary Figure 16. The miRNAs-mediated down-regulation of CIC levels contributes to the increase in cell proliferation in PC-3 cells.
Cell growth assay for the PC-3 cells treated with different combinations of control, three miRNAs or siCIC duplexes, and control or CIC-S-expressing lentivirus.Three independent experiments were carried out. **P<0.01. All error bars show s.e.m.

Supplementary Figure 17

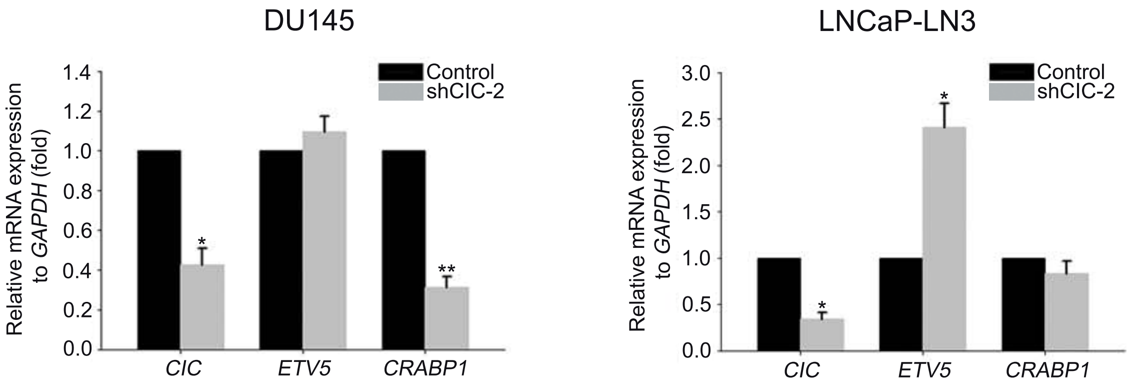

Supplementary Figure 17. Effect of CIC knock-down on expression of *ETV5* and *CRABP1* in DU145 and LNCaP-LN3 cells.

qRT-PCR analysis for *CIC*, *ETV5* and *CRABP1* levels in DU145 and LNCaP-LN3 cells that stably express shRNA for control or *CIC*. Three independent experiments were performed. *P<0.05 and **P<0.01. All error bars show s.e.m.

Supplementary Table 1. The number of non-cancerous prostate gland, PIN, and prostatic adenocarcinoma regions found in each section of 13 prostate cancer patient samples and the percentage of CIC^+ cells in prostate glands for each phenotype.

Patient #	Gleason score	Gland type	Number	% of CIC^+ cells (Average ± S.E)
1	7	Non-cancerous gland	30	34.8 ± 3.6
		PIN	315	11.1 ± 2.8
		Prostatic adenocarcinoma	5	2.8 ± 0.6
2	7	Non-cancerous gland	7	68.5 ± 4.7
		PIN	110	21.1 ± 1.7
		Prostatic adenocarcinoma	35	1.1 ± 0.7
3	N.A.	Non-cancerous gland	25	63.8 ± 5.1
		PIN	122	25.3 ± 1.3
		Prostatic adenocarcinoma	156	1.4 ± 0.6
4	7	Non-cancerous gland	91	72.5 ± 6.4
		PIN	168	21.1 ± 2.8
		Prostatic adenocarcinoma	8	5.1 ± 0.6
5	8	Non-cancerous gland	24	70.9 ± 0.4
		PIN	314	25.7 ± 2.8
		Prostatic adenocarcinoma	18	5.1 ± 0.5
6	7	Non-cancerous gland	51	74.4 ± 2.5
		PIN	265	36.2 ± 5.7
		Prostatic adenocarcinoma	82	3.4 ± 1.5
7	6	Non-cancerous gland	68	70.6 ± 5.9
		PIN	228	20.5 ± 2.7
		Prostatic adenocarcinoma	18	1.4 ± 1.4

8	5	Non-cancerous gland	85	66.6 ± 5.3
		PIN	353	28.2 ± 2.6
		Prostatic adenocarcinoma	18	1.7 ± 0.6
9	7	Non-cancerous gland	40	67.7 ± 3.5
		PIN	288	22.8 ± 2.1
		Prostatic adenocarcinoma	12	2.9 ± 1.3
10	7	Non-cancerous gland	72	64.2 ± 6.8
		PIN	235	26.1 ± 3.0
		Prostatic adenocarcinoma	7	5.2 ± 0.6
11	5	Non-cancerous gland	38	63.6 ± 3.0
		PIN	334	11.7 ± 4.7
		Prostatic adenocarcinoma	9	4.1 ± 1.9
12	8	Non-cancerous gland	58	85.9 ± 4.9
		PIN	193	11.6 ± 4.5
		Prostatic adenocarcinoma	9	1.3 ± 1.3
13	9	Non-cancerous gland	8	62.8 ± 7.3
		PIN	176	21.9 ± 2.6
		Prostatic adenocarcinoma	185	5.9 ± 2.1

Prostate tissue sections from 13 prostate cancer patients were stained with anti-CIC and anti-K8 antibodies and DAPI. The number of glands for each phenotype (non-cancerous, PIN, and prostatic adenocarcinoma) was counted under the ZEISS Axioplan2 microscope, and the percentage of cells with nuclear CIC expression (CIC^+ cells) was calculated using randomly selected 4~7 glands for each phenotype. N.A.: not analyzed.

Supplementary Table 2. Differentially expressed genes in CIC knock-down PC-3 cells.

Up-regulated genes in CIC knock-down PC-3 cells

Symbol	RPKM		FC (log2)	P-value
	Control	shCIC-3		
CRABP1	3.55	329.69	6.54	0.0003
FLT1	1.40	25.77	4.21	0.0001
IFI27	2.36	42.32	4.17	0.0159
SLAIN1	0.14	2.40	4.08	0.0387
PPP2R2C	0.23	3.45	3.90	0.0100
IFI6	43.65	561.65	3.69	0.0001
NUDT11	0.38	4.47	3.57	0.0233
CCNA1	0.82	8.04	3.29	0.0300
FAM107A	0.26	2.41	3.21	0.0332
HNF1B	0.47	4.30	3.20	0.0343
ABCG1	0.76	6.72	3.14	0.0081
IFITM1	25.35	221.34	3.13	0.0001
RGP1	273.32	2348.90	3.10	0.0004
TMPRSS15	0.50	4.13	3.05	0.0115
ZNF385A	0.91	7.30	3.00	0.0271
PRPH	0.55	4.38	2.99	0.0425
MSMP	451.60	3561.83	2.98	0.0258
CPLX1	0.70	5.10	2.87	0.0499
HR	0.51	3.66	2.84	0.0063
CREB3L1	1.18	8.36	2.82	0.0054
RAB31	3.95	27.47	2.80	0.0001
NAT1	0.47	3.14	2.74	0.0397
TMEM27	2.67	17.74	2.73	0.0099
OAS1	7.64	50.50	2.72	0.0002
NMB	10.50	68.39	2.70	0.0005
FCGR2A	0.81	5.26	2.70	0.0370
MX1	5.59	36.36	2.70	0.0001
HLF	1.17	7.56	2.70	0.0017
COL8A1	0.74	4.80	2.69	0.0220
FOXRED2	0.50	3.24	2.69	0.0249
IRF5	0.63	3.93	2.64	0.0493
MUC5B	1.24	7.68	2.63	0.0001
ACOX2	3.66	22.05	2.59	0.0012
SNX10	5.21	31.36	2.59	0.0006

**THTPA**	7.16	42.30	2.56	0.0029
**ANGPTL4**	0.99	5.69	2.52	0.0218
**NIPAL4**	0.64	3.65	2.50	0.0288
**ARSI**	0.58	3.25	2.49	0.0268
**H2BFM**	1.87	10.42	2.48	0.0128
**IFI44**	4.03	22.25	2.46	0.0034
**TUBB2A**	7.14	38.22	2.42	0.0010
**CDH3**	1.29	6.86	2.41	0.0096
**CSRP2**	15.99	83.28	2.38	0.0004
**POLE4**	165.29	830.30	2.33	0.0001
**RBM24**	1.32	6.47	2.29	0.0188
**IL22RA1**	0.76	3.67	2.27	0.0444
**CRABP2**	31.47	151.44	2.27	0.0001
**BHLHE41**	4.98	23.46	2.24	0.0003
**C1R**	3.43	15.93	2.22	0.0078
**MMP1**	12.68	58.90	2.22	0.0007
**SIDT1**	1.39	6.32	2.19	0.0041
**CABLES1**	9.25	41.78	2.17	0.0002
**HSD17B2**	9.55	42.63	2.16	0.0018
**SCARA5**	1.82	8.05	2.14	0.0045
**IFI44L**	1.69	7.43	2.14	0.0029
**FKBP3**	58.51	256.65	2.13	0.0007
**SPARC**	5.71	24.93	2.13	0.0004
**FAM131B**	1.42	6.17	2.12	0.0084
**EFNB3**	0.94	4.06	2.10	0.0289
**TSEN2**	10.94	46.80	2.10	0.0004
**YY1P2**	2.23	9.52	2.10	0.0342
**GMPR**	2.40	10.16	2.08	0.0291
**ZNF818P**	1.09	4.62	2.08	0.0291
**SOD3**	2.15	9.05	2.08	0.0289
**ZNF702P**	2.79	11.73	2.07	0.0062
**FGGY**	2.12	8.88	2.07	0.0325
**HIST1H2BH**	22.95	95.84	2.06	0.0196
**DDX58**	4.00	16.64	2.06	0.0007
**CETN2**	261.95	1088.40	2.05	0.0001
**CSRNP2**	3.74	15.44	2.05	0.0008
**CBY1**	19.20	78.61	2.03	0.0006
**PAK1**	2.67	10.89	2.03	0.0031
**MOXD1**	2.64	10.43	1.98	0.0067

**CDKN3**	222.90	873.58	1.97	0.0001
**DUSP16**	1.56	6.09	1.97	0.0059
**EPCAM**	11.91	46.34	1.96	0.0011
**DERA**	15.85	61.47	1.96	0.0011
**PARP9**	4.29	16.52	1.94	0.0249
**LOC100499489**	1.69	6.41	1.92	0.0481
**JAZF1**	0.97	3.69	1.92	0.0408
**KCTD18**	7.69	29.03	1.92	0.0013
**FBXL12**	16.21	60.46	1.90	0.0012
**PLEKHO1**	2.49	9.22	1.89	0.0474
**DDX60**	1.34	4.89	1.87	0.0120
**NPY1R**	5.30	19.13	1.85	0.0036
**UCP2**	3.37	12.12	1.85	0.0314
**ZNF518B**	1.19	4.24	1.83	0.0122
**C7orf25**	8.52	30.32	1.83	0.0040
**CLU**	7.03	24.97	1.83	0.0016
**ZNF668**	2.90	10.30	1.83	0.0214
**FAM127A**	490.80	1740.96	1.83	0.0020
**LHX6**	0.92	3.25	1.82	0.0491
**CSGALNACT1**	1.18	4.15	1.81	0.0325
**C1S**	2.50	8.74	1.81	0.0231
**MTHFD2L**	4.76	16.59	1.80	0.0099
**FGFBP1**	12.70	44.24	1.80	0.0043
**BRINP2**	7.56	26.23	1.79	0.0019
**CTSH**	23.54	81.25	1.79	0.0014
**OAS2**	5.07	17.44	1.78	0.0054
**AGR2**	41.57	142.92	1.78	0.0017
**SAV1**	8.28	28.43	1.78	0.0019
**LOC93622**	14.31	48.78	1.77	0.0028
**TMEM171**	4.51	15.28	1.76	0.0422
**FLVCR1-AS1**	11.54	39.07	1.76	0.0204
**PLA2G7**	5.72	19.32	1.76	0.0161
**TPD52L1**	9.11	30.70	1.75	0.0104
**PKIB**	22.33	74.63	1.74	0.0010
**CDKN2C**	9.69	32.23	1.73	0.0062
**CHURC1-FNTB**	65.58	218.12	1.73	0.0070
**SIPA1L2**	2.18	7.23	1.73	0.0068
**LRRC34**	3.30	10.94	1.73	0.0342
**IGFBP6**	52.31	172.11	1.72	0.0015

**CDC37L1**	3.21	10.55	1.72	0.0457
**C12orf10**	66.96	219.98	1.72	0.0007
**GJB3**	25.12	82.42	1.71	0.0012
**OPRL1**	1.35	4.41	1.71	0.0460
**GSKIP**	18.20	59.48	1.71	0.0013
**OASL**	4.26	13.88	1.70	0.0280
**TMEM251**	13.34	43.12	1.69	0.0120
**C3orf18**	33.98	108.84	1.68	0.0032
**CBS**	5.50	17.04	1.63	0.0106
**EPHB6**	1.60	4.94	1.63	0.0352
**VASH1**	1.01	3.14	1.63	0.0328
**SAMD9**	3.18	9.82	1.63	0.0047
**PPL**	1.82	5.61	1.62	0.0126
**ARID2**	2.33	7.13	1.61	0.0043
**PDLIM1**	152.95	467.66	1.61	0.0018
**PRICKLE1**	2.98	9.09	1.61	0.0153
**MACRCH9**	3.15	9.59	1.61	0.0280
**CTGF**	40.27	121.97	1.60	0.0015
**USP10**	31.09	93.56	1.59	0.0018
**GLI3**	2.89	8.68	1.59	0.0064
**COX7C**	3732.25	11222.80	1.59	0.0049
**DHRS2**	16.32	49.04	1.59	0.0048
**PEX11B**	40.68	121.60	1.58	0.0011
**LINC01116**	16.38	48.72	1.57	0.0142
**SAYSD1**	3.79	11.28	1.57	0.0433
**PLSCR1**	15.63	46.47	1.57	0.0043
**WDR61**	101.43	297.72	1.55	0.0022
**PDGFA**	3.37	9.81	1.54	0.0231
**CDK2AP1**	276.48	796.72	1.53	0.0023
**AK5**	2.61	7.51	1.52	0.0359
**SRD5A1**	3.60	10.28	1.51	0.0325
**KRT7**	64.93	185.39	1.51	0.0032
**LINC00263**	26.04	73.95	1.51	0.0088
**BATF3**	0.00	4.64	+inf	0.0001
**OR4C3**	0.00	2.29	+inf	0.0004
**RPRML**	0.00	2.29	+inf	0.0001
**SNORD32A**	0.00	898.24	+inf	0.0484
**BST2**	0.00	13.11	+inf	0.0001
**KLK5**	0.00	3.80	+inf	0.0001

**ZSCAN18**	0.00	2.10	+inf	0.0001
**C20orf141**	0.00	4.17	+inf	0.0002
**LINC01432**	0.00	2.15	+inf	0.0059
**SLC51A**	0.00	3.70	+inf	0.0001
**RUNDC3B**	0.00	2.67	+inf	0.0001

Down-regulated genes in CIC knock-down PC-3 cells

**Symbol**	**RPKM**		**FC (log2)**	**P-value**
	**Control**	**shCIC-3**		
**GHRL**	2.86	0.09	-5.03	0.0332
**SERPINB2**	17.61	0.97	-4.19	0.0023
**ARHGAP22**	12.27	0.91	-3.76	0.0031
**CXCL5**	405.78	33.84	-3.58	0.0001
**PDPN**	15.61	1.57	-3.32	0.0012
**CST1**	31.60	3.52	-3.17	0.0105
**MT1X**	487.97	56.24	-3.12	0.0001
**DYSF**	7.38	0.98	-2.92	0.0008
**SLAMF9**	10.88	1.44	-2.91	0.0330
**GRK4**	2.54	0.36	-2.83	0.0386
**YJEFN3**	19.28	2.77	-2.80	0.0169
**UAP1L1**	7.86	1.22	-2.69	0.0107
**GOLGA8A**	2.82	0.48	-2.55	0.0211
**TMEM161B-AS1**	54.83	9.43	-2.54	0.0082
**DUSP10**	3.62	0.63	-2.51	0.0193
**IL33**	47.21	8.34	-2.50	0.0001
**CDKL5**	3.74	0.68	-2.46	0.0374
**TGFBI**	32.97	6.03	-2.45	0.0004
**ABHD16A**	5.04	0.93	-2.44	0.0223
**HMOX1**	14.74	2.71	-2.44	0.0170
**PIDD1**	11.23	2.16	-2.38	0.0057
**SERTAD1**	11.67	2.27	-2.36	0.0363
**CLCN6**	3.48	0.69	-2.34	0.0465
**SOX21**	7.23	1.43	-2.34	0.0201
**ARHGDIB**	11.75	2.32	-2.34	0.0390
**CSF2**	68.71	14.15	-2.28	0.0028
**ADAMTS1**	37.10	7.67	-2.27	0.0001
**SCNN1D**	4.67	0.97	-2.27	0.0177
**SNAI2**	22.69	4.74	-2.26	0.0012

**TFF2**	288.29	61.18	-2.24	0.0001
**SDHAP1**	3.50	0.75	-2.21	0.0399
**TENM3**	3.65	0.79	-2.21	0.0013
**GAS6**	11.72	2.56	-2.20	0.0112
**ID3**	29.56	6.66	-2.15	0.0086
**ZDHHC8**	6.75	1.55	-2.12	0.0051
**LOC100288069**	6.28	1.48	-2.09	0.0478
**PLXNB3**	3.32	0.80	-2.06	0.0196
**WEE2-AS1**	5.35	1.30	-2.05	0.0275
**OSBPL7**	8.72	2.11	-2.04	0.0056
**MYL5**	26.66	6.51	-2.03	0.0370
**IL13RA2**	20.86	5.10	-2.03	0.0107
**RALGPS1**	3.67	0.90	-2.03	0.0492
**CXCL3**	295.65	73.32	-2.01	0.0001
**FXN**	7.31	1.85	-1.99	0.0253
**RPLP0P2**	19.34	4.93	-1.97	0.0011
**TMEM52**	13.98	3.74	-1.90	0.0428
**PEX26**	7.29	1.95	-1.90	0.0100
**TBC1D2**	4.66	1.25	-1.90	0.0472
**ABCC10**	2.95	0.79	-1.89	0.0432
**NBPF9**	7.99	2.16	-1.89	0.0190
**MT2A**	8700.77	2379.54	-1.87	0.0018
**EPGN**	9.51	2.65	-1.84	0.0173
**ADAM12**	6.83	1.93	-1.82	0.0101
**HCLS1**	32.73	9.26	-1.82	0.0027
**LPIN3**	6.95	1.98	-1.81	0.0205
**LRIG1**	4.32	1.24	-1.80	0.0481
**TRIM46**	3.59	1.04	-1.79	0.0489
**ROBO4**	3.93	1.14	-1.78	0.0311
**CSF3**	101.72	30.27	-1.75	0.0009
**GBX2**	12.57	3.74	-1.75	0.0361
**HIST1H2AC**	50.01	15.33	-1.71	0.0437
**ZFYVE27**	12.01	3.72	-1.69	0.0102
**VNN1**	9.05	2.80	-1.69	0.0146
**MED22**	11.94	3.72	-1.68	0.0202
**GPR4**	40.70	12.91	-1.66	0.0014
**COQ4**	14.44	4.58	-1.66	0.0334
**HAS2**	8.12	2.58	-1.65	0.0219
**DEPDC1B**	21.42	6.82	-1.65	0.0057

**VCAN**	7.62	2.44	-1.64	0.0113
**CXCL8**	426.65	136.77	-1.64	0.0017
**LOC146880**	5.84	1.89	-1.63	0.0452
**SHH**	19.69	6.51	-1.60	0.0199
**SLC37A2**	8.43	2.79	-1.59	0.0145
**MROH1**	16.50	5.60	-1.56	0.0133
**LCP1**	37.25	12.66	-1.56	0.0029
**SGK1**	15.29	5.20	-1.56	0.0159
**GALNT18**	12.92	4.41	-1.55	0.0146
**RPPH1**	719.80	245.71	-1.55	0.0045
**CEBPD**	31.15	10.78	-1.53	0.0171
**DUSP7**	28.98	10.04	-1.53	0.0065
**BOK**	19.42	6.74	-1.53	0.0102
**PDGFD**	4.43	1.54	-1.52	0.0462
**MED18**	11.53	4.04	-1.51	0.0498
**COL6A3**	39.64	13.92	-1.51	0.0012
**CCDC15**	9.52	3.36	-1.50	0.0236
**LRRC38**	61.43	0.00	- inf	0.0001
**FLJ27354**	3.07	0.00	- inf	0.0007
**LOC728989**	2.66	0.00	- inf	0.0005
**SNORA52**	225.64	0.00	- inf	0.0268
**CLLU1OS**	5.12	0.00	- inf	0.0006
**PLA2G1B**	2.90	0.00	- inf	0.0057
**MT1L**	12.43	0.00	- inf	0.0001
**SNORA64**	101.54	0.00	- inf	0.0487
**MT1G**	2.72	0.00	- inf	0.0474
**SNORA75**	165.90	0.00	- inf	0.0304
**HAO1**	2.43	0.00	- inf	0.0001
**WFDC10B**	5.20	0.00	- inf	0.0037
**MIR570**	409.49	0.00	- inf	0.0073
**IQCF1**	3.98	0.00	- inf	0.0003
**VTRNA1-2**	436.17	0.00	- inf	0.0474
**HIST1H4A**	6.20	0.00	- inf	0.0057
**MIR222**	341.93	0.00	- inf	0.0057
**BEX5**	5.96	0.00	- inf	0.0001

Genes are listed when satisfied with the following criteria: FC (log2) > 2, P-value < 0.05 and RPKM-difference > 2. RPKM, reads per kilobase per million mapped reads; FC, fold change; +inf, Infinite (The RPKM value in control PC-3 cells is zero); -inf, Infinite (The RPKM value in shCIC-3 PC-3 cells is zero).

Supplementary Table 3. Raw data for clonogenic, invasion, BrdU labeling, and wound healing assays.

1) Clonogenic assay

PC-3	Control	CIC-S	CIC-L
Average ± s.e	147.0±6.6	60.0±9.2	34.0±11.0
Analyzed cell number	588	240	136

PC-3	Control	shCIC-1	shCIC-2	shCIC-3
Average ± s.e	81.0±6.9	132.0±12.1	147.5±12.4	199.0±25.9
Analyzed cell number	648	1056	1180	1592

LNCaP	Control	shCIC-1	shCIC-2	shCIC-3
Average ± s.e	76.0±9.3	151.8±12.3	184.0±22.0	120.2±11.0
Analyzed cell number	457	911	1104	721

PC-3	Control	FLAG-CRABP1
Average ± s.e	31.2±1.6	65.0±2.7
Analyzed cell number	656	1366

PC-3	Control virus			CIC-S overexpressing virus		
	Control	93/106b/375	siCIC	Control	93/106b/375	siCIC
Average ± s.e	39.0±2.7	82.8±5.5	85.0±7.2	32.0±3.5	54.3±4.3	47.8±4.3
Analyzed cell number	234	496	510	192	326	287

2) Invasion assay

PC-3	Control	CIC-S	CIC-L
Average ± s.e	161.0±14.5	67.2±8.0	59.0±6.4
Analyzed cell number	4670	1961	1180

PC-3	Control	shCIC-1	shCIC-2	shCIC-3
Average ± s.e	67.6±6.9	216.2±13.1	233.1±14.7	262.8±11.7
Analyzed cell number	1488	6296	6761	9725

LNCaP	Control	shCIC-1	shCIC-2	shCIC-3
Average ± s.e	53.6±9.5	118.9±23.9	163.5±16.0	126.2±15.5
Analyzed cell number	697	1784	3271	2651

PC-3	Control	FLAG-CRABP1
Average ± s.e	57.4±4.6	129.5±11.5
Analyzed cell number	4305	8550

PC-3	Control virus			CIC-S overexpressing virus		
	Control	93/106b/375	siCIC	Control	93/106b/375	siCIC
Average ± s.e	33.9±3.6	113.8±9.5	114.9±8.7	27.2±3.8	44.3±4.2	38.8±5.0
Analyzed cell number	1085	3641	4252	843	1506	1359

3) BrdU labeling assay

PC-3	Control	CIC-S	CIC-L
Average (%) ± s.e	44.3.0±4.1	10.7±2.5	3.1±0.6
Analyzed cell number	3076	1568	1649

PC-3	Control	shCIC-1	shCIC-2	shCIC-3
Average (%) ± s.e	24.7±1.8	46.1 ±2.1	47.9±2.9	48.0±2.5
Analyzed cell number	999	1925	2506	2084
LNCaP	Control	shCIC-1	shCIC-2	shCIC-3
Average (%) ± s.e	23.5±1.0	39.5±2.3	45.3±1.5	37.6±2.2
Analyzed cell number	3647	4692	5350	7322

4) Wound healing assay

PC-3	Control		CIC-S		CIC-L	
Incubation time (h)	0	84	0	84	0	84
Average ± s.e	14±3	250±14	10±2	144±10	5±1	80±5
Analyzed cell number	391	6759	284	3912	137	2151

PC-3	Control		shCIC-1		shCIC-2		shCIC-3	
Incubation time (h)	0	24	0	24	0	24	0	24
Average ± s.e	11±1	42±4	10±1	216±19	7±1	195±14	8±1	344±18
Analyzed cell number	290	1221	186	3890	167	4108	233	10000
